# Interferon-induced miR-7705 modulates the anti-virus activity of cholesterol 25-hydroxylase

**DOI:** 10.1128/jvi.01198-25

**Published:** 2025-09-10

**Authors:** Le Wang, Hongxiao Song, Fengchao Xu, Yujia Zhu, Mian Huang, Jing Xu, Xiaolu Li, Fei Wang, Fan Yang, Yang Lei, Pujun Gao, Guangyun Tan

**Affiliations:** 1 Department of Hepatology, Center of Infectious Diseases and Pathogen Biology, Institute of Translational Medicine, The First Hospital of Jilin University 117971, Changchun, Jilin, China; 2 Jilin Provincial Key Laboratory of Metabolic Liver Diseases, Jilin University 12510, Changchun, Jilin, China; 3 China-Singapore Belt and Road Joint Laboratory on Liver Disease Research, Changchun, Jilin, China; 4 Health Examination Center, The First Hospital of Jilin University 117971, Changchun, Jilin, China; 5 Department of Pediatrics, the First Hospital, Jilin University 12510, Changchun, Jilin, China; 6 Department of Anesthesiology, the First Hospital, Jilin University 12510, Changchun, Jilin, China; Wake Forest University School of Medicine, Winston-Salem, North Carolina, USA

**Keywords:** CH25H, miR-7705, HBV replication, antiviral regulation, IFN-α

## Abstract

Cholesterol 25-hydroxylase (CH25H), an interferon-stimulated gene (ISG), has been implicated in broad-spectrum antiviral immunity. Here, we identify CH25H as a potent suppressor of hepatitis B virus (HBV) replication that significantly outperforms IFN-α in reducing HBV DNA, pregenomic RNA (pgRNA), HBsAg, and HBeAg, without inducing cytotoxicity. However, CH25H is weakly expressed in hepatocytes and only modestly induced by type I interferon. We found that CH25H expression is tightly controlled by microRNAs, especially miR-7705, which is induced by IFN-α in a STAT1-dependent manner. miR-7705 directly targets the 3′UTR of CH25H, suppressing its expression and enhancing HBV replication. Knockdown of miR-7705 restores CH25H levels, enhances IFN-induced antiviral responses, and suppresses HBV replication in both transient transfection and infection models. Mechanistically, miR-7705 overexpression nullifies CH25H-mediated suppression of HBV, whereas this effect is abolished in CH25H-knockout cells, confirming the specificity of the miR-7705–CH25H axis. Furthermore, we demonstrate that this regulatory axis also governs CH25H-mediated restriction of RNA viruses, including EV71 and CVB3, suggesting its broad antiviral relevance. Importantly, antagonizing miR-7705 enhances the efficacy of IFN-based therapy against both DNA and RNA viruses. These findings reveal miR-7705 as a negative regulator of CH25H and position the miR-7705–CH25H axis as a promising target to improve antiviral immunity.

**IMPORTANCE:**

This study highlights the critical role of miR-7705 in regulating the antiviral effects of interferon (IFN) therapy, particularly in the context of chronic HBV infection. By identifying miR-7705 as a key modulator of CH25H, a protein essential for controlling HBV replication, our research provides new insights into the mechanisms that limit the effectiveness of IFN treatment. Targeting miR-7705 could improve the efficacy of IFN-based therapies, offering a potential strategy to better manage HBV and other viral infections. This research paves the way for developing adjunctive treatments that enhance the body's natural antiviral responses.

## INTRODUCTION

Hepatitis B virus (HBV) infection significantly impacts global health, leading to acute and chronic liver diseases such as liver failure, cirrhosis, and hepatocellular carcinoma (HCC). HBV, a partially double-stranded DNA virus, forms covalently closed circular DNA (cccDNA) in hepatocytes, serving as a stable template for viral replication. Current antiviral therapies include nucleos(t)ide analogs (NAs) and pegylated interferon alpha (PEG-IFN-α) (1). Although NAs effectively suppress HBV replication, achieving a functional cure is rare. In contrast, PEG-IFN-α can lead to sustained virologic control after treatment cessation, although its overall efficacy is limited due to persistent IFN resistance (2).

Interferons (IFNs), discovered in 1957, are crucial components of the innate immune response. Upon viral infection, type I IFNs (α, β, etc.) play a major role in initiating the Janus kinase (JAK)-signal transducer and activator of transcription (STAT) pathway and inducing numerous interferon-stimulated genes (ISGs), including Cholesterol 25-hydroxylase (CH25H). This pathway is essential for mounting an effective antiviral response (3, 4). This response includes both protein-coding and non-coding genes with antiviral properties. Recent studies have highlighted the role of interferons (IFNs) in regulating microRNAs (miRNAs), which, along with proteins, contribute significantly to antiviral defense (5). However, the effectiveness of IFN therapy is often limited, as many patients exhibit suboptimal responses—possibly due to certain interferon-stimulated genes (ISGs) functioning as negative regulators of HBV (6). Therefore, elucidating the mechanisms underlying IFN-induced antiviral responses remains essential for improving therapeutic outcomes.

Cholesterol 25-hydroxylase (CH25H), located in the endoplasmic reticulum, catalyzes the conversion of cholesterol to 25-hydroxycholesterol (25HC) (7). Both CH25H and 25HC exhibit comprehensive antiviral activity (8, 9), impacting viral entry, penetration, and replication through lipid metabolism, immune modulation, and direct interference with viral components. Our previous work showed that CH25H inhibits HBV replication by blocking the nuclear translocation of HBx (10), a key transcriptional factor for HBV replication (11). This inhibitory effect occurs independently of CH25H’s enzymatic activity. Despite these findings, the regulatory mechanisms controlling CH25H expression are unclear, prompting further investigation. Studies show CH25H can be induced by toll-like receptor (TLR) agonists, such as TLR4 and TLR3, in macrophages (12).

MicroRNAs (miRNAs) are non-coding RNAs present in various biological contexts, including bodily fluids (5, 13). They typically suppress gene expression through RNA silencing. Once incorporated into the RNA-induced silencing complex (RISC) (14), a single miRNA binds to target mRNAs, leading to translation inhibition. Several miRNAs have demonstrated direct antiviral activity (15, 16), with some shown to enhance HBV replication (17), raising questions about their role in regulating CH25H-mediated HBV inhibition.

In this study, we initially focus on investigating the effects of miR-7705 and CH25H on HBV replication. Our findings reveal that miR-7705 binds to the 3' UTR of CH25H, reducing its mRNA levels and counteracting CH25H’s inhibitory effect on HBV replication. Additionally, we observed that miR-7705 similarly influences the replication of EV71 and CVB3. In contrast to HBV, a DNA virus, both EV71 and CVB3 are single-stranded, positive-sense RNA viruses; EV71 causes hand, foot, and mouth disease and is associated with complications in the central nervous system (18–20), whereas CVB3 is linked to myocarditis and dilated cardiomyopathy due to its cytopathic effects on heart tissue and may contribute to type 1 diabetes through immune-mediated pancreatic damage (21, 22). Given that both miR-7705 and CH25H are interferon-stimulated genes (ISGs), these findings provide insights into potential strategies to overcome interferon resistance in antiviral therapies.

## MATERIALS AND METHODS

### Sample collection

This study enrolled 28 HBV-infected patients and 18 healthy controls (Tables S1 and S2). Venous blood samples (5 mL) were collected to extract serum and peripheral blood mononuclear cells (PBMCs). HBV DNA levels were measured using Roche’s COBAS TaqMan Kit, and liver function, along with biochemical parameters, was assessed using an automatic biochemical analyzer.

### Cell culture, plasmids, and reagents

Human embryonic kidney 293T (HEK293T), Caco2, HepG2, Huh7, and WRL68 cells were cultured in Dulbecco’s-modified Eagle medium (DMEM). Both media contained 10% heat-inactivated fetal bovine serum, 100 IU/mL penicillin, and 100 mg/mL streptomycin, with incubation at 37°C under 5% CO2. The CH25H wild-type expression plasmids were constructed previously (10). For CH25H 3' UTR constructs, the sequence complementary to miR-7705 binding on the 3' UTR of CH25H was inserted into the pmirGLO vector to create CH25H 3' UTR WT. A mutated binding site sequence was similarly inserted to generate CH25H 3' UTR Mut. Reporter plasmids for miR-7705, miR-7-5P, and novel-miR-839 were generated by inserting complementary binding sequences into the pmirGLO vector. The miR-7705 sponge overexpression vector was constructed by inserting a sequence with the reverse complementary mature miR-7705 sequence (preserving the seed region) and mutating the RISC-binding site into the VR1012 vector. All ISG plasmids were generated by cloning the corresponding coding sequences into the VR1012 vector with either a Flag or HA tag and are currently archived in our laboratory. Primer sequences used for PCR are provided in Table S3.

### miRNA sequencing and differential expression analysis

Total RNA was extracted from four groups of HepG2 cells (untreated or treated with IFN-α [20  ng/mL] for 3, 8, or 24  h), each with three biological replicates, using TRIzol Reagent (Life Technologies). miRNA sequencing was performed by Beijing Biomarker Technologies Co. using the Hieff NGS Ultima Dual-mode mRNA Library Prep Kit (Yeasen Biotechnology, Shanghai) and the Illumina NovaSeq platform. Raw reads were processed and aligned to the Silva, GtRNAdb, Rfam, and Repbase databases using Bowtie and mapped to the Homo sapiens GRCh38_release95 reference genome via the BMKCloud platform. Known miRNAs were identified by aligning reads to miRBase v22 (allowing one mismatch in the flanking regions), and novel miRNAs were predicted using miRDeep2. Differential expression was analyzed with thresholds of |log₂(fold change)| ≥0.58 and *P*  ≤  0.05. The Benjamini-Hochberg method was used to adjust *P*-values, and the false discovery rate (FDR) was used to identify significantly altered miRNAs.

### Western blotting (WB)

Cells were harvested 24–48 h after transfection with expression plasmids and lysed using a buffer containing 50  mM Tris-HCl (pH 8.0), 150  mM NaCl, and 1% NP-40, supplemented with protease inhibitors (Roche, USA). Immunoblotting was conducted as described previously (23). Protein concentration was determined using the Coomassie Plus Protein Assay Kit (Thermo Scientific, Rockford, IL, USA). Equal amounts of protein were separated by SDS-PAGE and transferred onto PVDF membranes. Membranes were blocked with 5% skim milk in TBS containing 0.1% Tween-20 (TBS-T) and incubated with the indicated primary antibodies. Rabbit polyclonal anti-CH25H (1:1000; Catalog #05283, Novus Biologicals), Rabbit polyclonal anti-HA (1:1000; Proteintech), Rabbit polyclonal anti-HBx (a kind gift from Simon Fletcher and Rudolf Beran, Gilead Sciences), and Anti-HBc (a kind gift from Prof. Bin Ju, Shenzhen Third People’s Hospital. Protein bands were visualized and quantified using the ChemiDoc XRS + Imaging System and software (Bio-Rad, Philadelphia, PA, USA).

### Quantitative real-time PCR

HBV RNAs, HBV DNA, and miR-7705 expression levels were quantified by SYBR Green-based real-time PCR. Reverse transcription for mRNA was performed using the First-Strand cDNA Synthesis Kit (TransGen, Beijing, China) with gene-specific and oligo dT primers. miR-7705 was reverse-transcribed using the All-in-One miRNA qRT-PCR and the miRNA First Strand cDNA Synthesis Kit (Sangon Biotech). Viral DNA was extracted from 200 µL of virus supernatant using the EasyPure Viral DNA/RNA kit (TransGen Biotech, Beijing, China, Cat. # ER201-01), following the manufacturer’s protocol. The extracted DNA was then subjected to qPCR analysis, and HBV DNA copies were quantified as previously reported (10). Primer sequences for qPCR are available in Table S4.

### ELISA

The supernatant from cells transfected with pHBV1.2 plasmids, infected with HBV, or cultured HepAD38 cells was collected for the detection of HBsAg and HBeAg levels using enzyme-linked immunosorbent assay (ELISA), according to the manufacturer’s instructions (Kehua Bioengineering, Shanghai, China).

### CRISPR/Cas9 knockout

HepG2 or 293T cells were seeded in 12-well plates and co-transfected with plasmids expressing Cas9, sgRNA, and a puromycin selection marker 16 h later. After 36 h, puromycin (2 µg/mL) was added for cell selection to eliminate untransfected cells. After 72 h of selection, live cells were sorted into 96-well plates at one cell per well for clonal selection. Each clone was monitored and expanded for further validation using immunoblotting and DNA sequencing to confirm gene editing efficiency. sgRNA sequences are listed in Table S5.

### Luciferase reporter assay

For microRNA reporter assays (24), HepG2 cells were plated in 24-well plates at a density of 1 × 10^5 cells per well and transfected with 1 µg of microRNA reporter plasmids using Lipofectamine 3000, following the manufacturer’s protocol. Transfection efficiency was assessed using a Renilla luciferase control plasmid. After 8 h, the cells were treated with IFNα (20 ng/mL) or PBS (control) for 24 h. For miR-7705 target identification, HepG2 cells were co-transfected with 0.5 µg CH25H 3' UTR WT or Mut plasmids and miR-NC or miR-7705 (40 nM or 80 nM) for 30 h. Firefly and Renilla luciferase activities were measured using the Dual-Luciferase Reporter Assay System (Promega).

### Immunofluorescence

HepG2 cells were transfected with Flag-HBx plasmids, HepG2 NTCP cells were infected with HBV, or HepAD38 cells were used. The cells were then fixed with 0.4% polyformaldehyde at room temperature (RT) for 10 min. Following fixation, the cells were washed with phosphate-buffered saline (PBS) and blocked with 5% bovine serum albumin (BSA) in PBS-Triton X-100 (PBST) for 30 min at 37°C. The cells were then incubated with HBx (Rabbit) or HBc (human) antibodies at 37°C for 1 h, washed with PBST, and subsequently incubated with goat anti-rabbit IgG conjugated to fluorescein Cy3 or rhodamine (TRITC, Human) (Proteintech). After washing with PBST, the cells were incubated with DAPI (1:500) for 5 min at RT. Finally, the cells were observed using fluorescence microscopy or a laser confocal microscope, and the images were analyzed using NIS Elements Viewer.

### CCK-8 assay for cell viability assessment

HepG2 cells were seeded into 96-well plates at a density of approximately 3,000 cells per well in 100  µL of complete medium. The plates were pre-incubated at 37°C in a humidified incubator with 5% CO₂ for 24 h to allow for cell attachment. Test compounds at various concentrations were then added to each well—either during cell seeding or after attachment, depending on the experimental design—with five replicate wells per concentration. The control group received an equivalent volume of DMSO. After incubation for 24, 48, or 72 h, cell viability was assessed using the Cell Counting Kit-8 (Yeasen Biotechnology, Shanghai) following the manufacturer’s instructions. The optical density (OD) at 450  nm was measured using a microplate reader. Cell viability and inhibition rates were calculated based on the absorbance values.

### HBV infection assay

The supernatant from HepAD38 cells was collected, and the virus was concentrated using PEG-it Virus Precipitation Solution (System Biosciences, USA). Lentiviral vectors expressing si-miR-7705 or si-scramble (lot# 250225LVL203 and 250228LVL208) were purchased from Hysigen Bioscience Co., Ltd. HepG2-NTCP cells were infected with the concentrated, serum-derived HBV at a multiplicity of infection (MOI) of 700 genome equivalents (Geq) per cell. Infection was carried out in the presence of 4% polyethylene glycol 8000 and 2.5% dimethyl sulfoxide (DMSO) for 24 h. Following infection, the cells were washed four times with PBS and cultured in DMEM for an additional 9 days. During this post-infection period, the cells were treated with or without interferon-α (IFN-α, 20 ng/mL) for 24 h prior to each medium change. At the end of the infection period, both the supernatant and cells were collected for analysis. HBV DNA, pregenomic RNA (pgRNA), HBsAg, and HBeAg levels were quantified using qPCR and ELISA. The infection efficiency was obtained by analyzing the positive rate of HBc using NIS Elements Viewer (out of 397 DAPI-positive cells, 113 were HBc-positive, resulting in an infection rate of 28.5%, Fig. S6A).

### Lentiviral-mediated knockdown of miR-7705

All lentiviral constructs, including those expressing si-miR-7705 and si-scramble, were obtained from Hysigen Bioscience Co., Ltd. Lentiviral transduction was performed at a multiplicity of infection (MOI) of 10 in HepAD38, HepG2-NTCP, HepG2, Huh7, and Caco-2 cells. Successful transduction was confirmed by the appearance of green fluorescence under an inverted fluorescence microscope (×200). The efficiency of lentivirus-mediated gene silencing was subsequently validated by qPCR and western blotting.

### EV71 and CVB3 infection

EV71 and CVB3 virus, kindly provided by Prof. Baisong Zheng of Jilin University was titrated by plaque assay (25). HepG2 cells were seeded in 12-well plates and infected with EV71 or CVB3 either directly or post-transfection with microRNA mimics or plasmids.

### Quantification and statistical analyses

Data analysis was performed using GraphPad Prism 10 software (GraphPad Software, San Diego, CA). A two-tailed unpaired *t*-test was used to assess the differences between groups, with a significance threshold of *P* < 0.05.

## RESULTS

### CH25H strongly inhibits HBV replication

**Fig 1:**
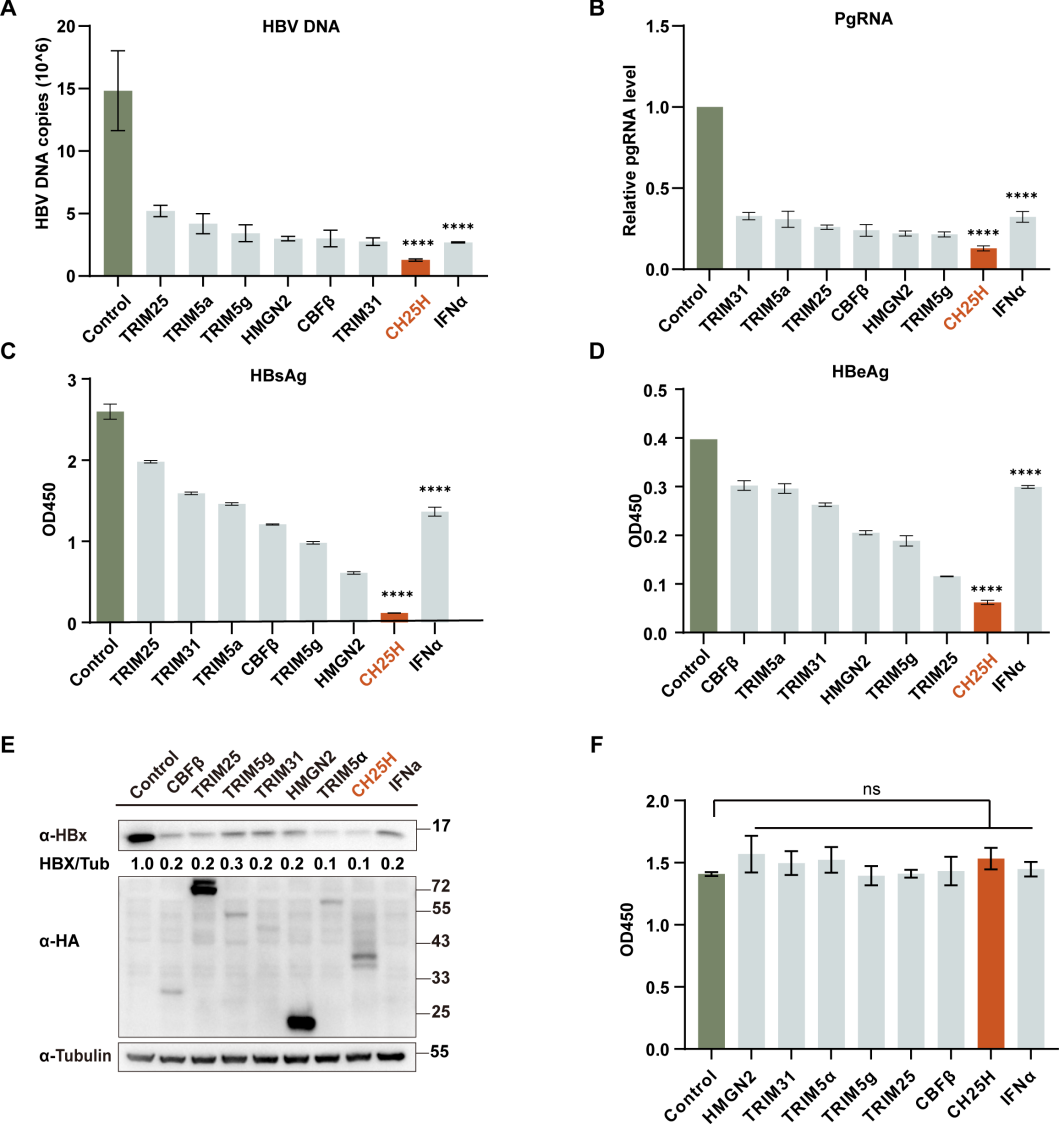
CH25H suppresses HBV replication. (**A–E**) HepG2 cells were co-transfected with a HA-tagged CH25H plasmid, other HA-tagged interferon-stimulated gene (ISG) expression plasmids, or an empty vector, along with the pHBV1.2 plasmid. At 24 h post-transfection, the cells were treated with PBS or IFN-α (20 ng/mL) for an additional 24 h. The cells were then harvested and analyzed by qPCR and western blot to assess HBV DNA copies and pregenomic RNA (pgRNA) levels (**A–B**), as well as HBx protein expression (**E**). Culture supernatants were collected to measure HBsAg and HBeAg levels via ELISA (**C–D**). OD450 refers to the optical density measured at 450 nm. Data are presented as the mean ± SD from three independent experiments. (**F**) Cell viability assay. HepG2 cells were treated as described in (**A–E**), and cell viability was assessed using the CCK-8 assay. Mean ± SD values from three independent experiments are shown. Statistical significance: *P  <  0.05; **P  <  0.01; ***P  <  0.001; ****P  <  0.0001, ns >0.05.

CH25H exhibits broad-spectrum antiviral activity and plays a critical role in host defense against viral infections. In our previous study, we identified CH25H as a potent anti-HBV effector that suppresses viral replication by interfering with the nuclear translocation of the hepatitis B virus X protein (HBx), thereby disrupting its transcriptional regulatory functions (10). To further evaluate the relative antiviral potency of CH25H, we systematically compared its activity with several other interferon-stimulated genes (ISGs) previously identified in our anti-HBV candidate list (10, 26–30). Remarkably, CH25H consistently exhibited the most robust inhibitory effects across multiple HBV markers, including HBV DNA, pregenomic RNA (pgRNA), hepatitis B surface antigen (HBsAg), and hepatitis B e antigen (HBeAg), significantly outperforming the classical antiviral cytokine IFN-α in all assays (Fig. 1A through D). In addition, CH25H markedly reduced intracellular HBx protein levels (Fig. 1E). Interestingly, although all tested ISGs similarly suppressed HBx expression, their overall effects on HBV replication and antigen production differed, likely due to distinct mechanisms of action beyond HBx degradation. Notably, the potent antiviral effects of all the ISGs we used were not associated with any detectable cytotoxicity, as cell viability remained unaffected following either plasmid transfection or IFN-α treatment, as assessed by viability assays (Fig. 1F). However, it is notable that CH25H exhibits low expression levels in hepatocytes and is challenging to induce with type I IFN (8), motivating our investigation into its regulatory mechanisms.

### Regulation of CH25H expression by microRNAs

**Fig 2:**
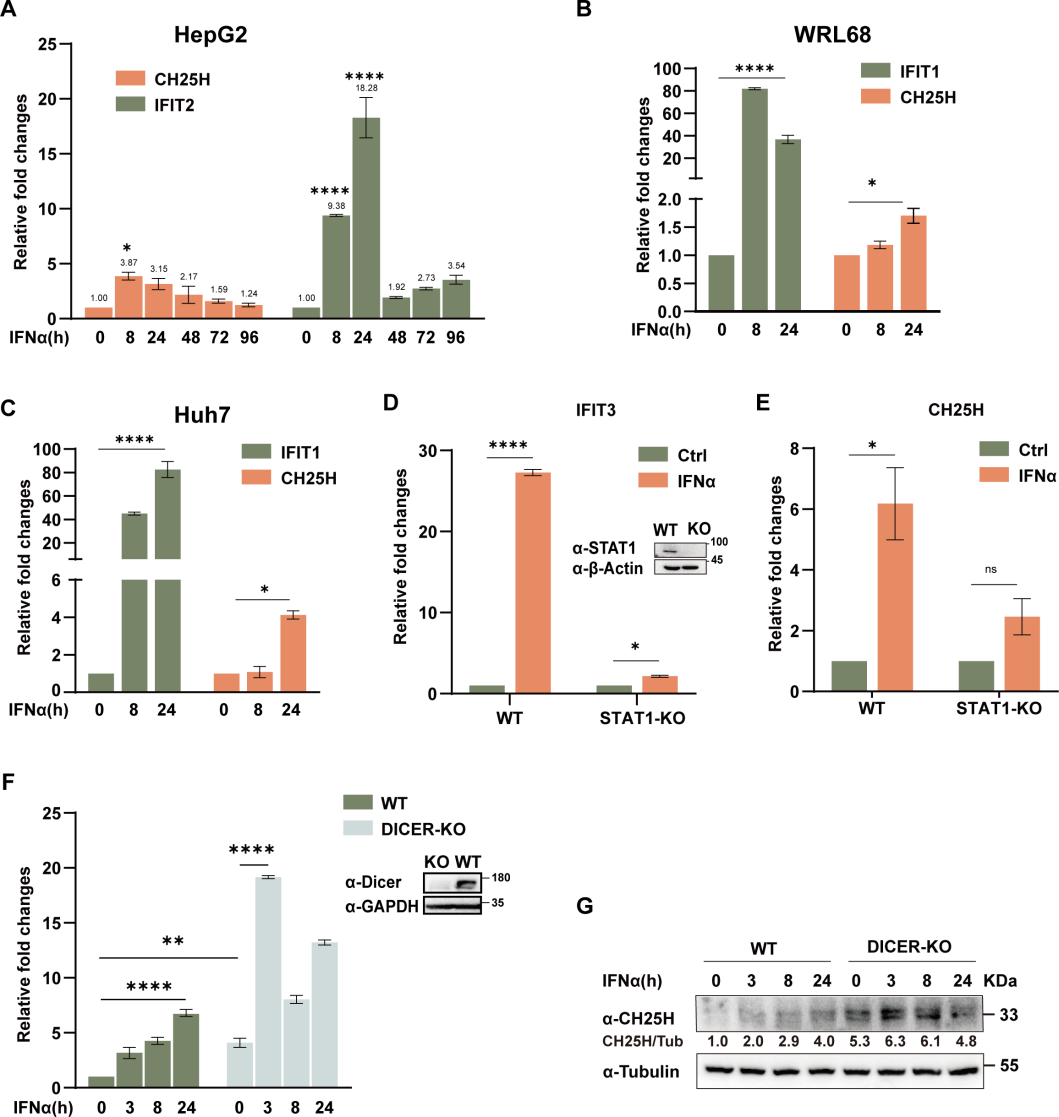
CH25H is induced by IFN-α in a STAT1- and DICER-dependent manner. (**A–C**) HepG2, WRL68, and Huh7 hepatic cell lines were treated with IFN-α (20 ng/mL) or left untreated, as indicated. Cells were harvested for qPCR analysis to evaluate the expression of CH25H and IFIT1 (or IFIT2). (**D–E**) Wild-type (WT) and STAT1 knockout (KO) HepG2 cells were treated with IFN-α (20 ng/mL) for the indicated durations, followed by qPCR to quantify CH25H and IFIT3 expression. Cell lysates were analyzed by immunoblotting using antibodies against STAT1 and β-actin. (**F**) WT and DICER KO 293T cells were treated with IFN-α (20 ng/mL) or left untreated as indicated. Cells were harvested for qPCR analysis of CH25H mRNA expression. Lysates were subjected to immunoblotting using anti-DICER and anti-GAPDH antibodies. (**G**) 293 T WT and DICER KO cells were treated as in panel (**F**), then harvested for immunoblotting to assess CH25H protein expression, with tubulin used as a loading control. Data represent the mean ± SD from three independent experiments. Statistical significance: *P  <  0.05; **P  <  0.01; ***P  <  0.001; ****P  <  0.0001, ns >0.05.

Given CH25H’s low expression in hepatocytes, we next sought to investigate its transcriptional and post-transcriptional regulation. Gene expression is regulated by various mechanisms, including DNA methylation, histone modification, and non-coding RNA activity, among others. Under normal physiological conditions, CH25H expression remains consistently low across most tissues (8). In this study, we further validated this finding in multiple cell lines, including HepG2, WRL68, and Huh7, where CH25H expression exhibited only a slight increase in response to IFN-α stimulation, in contrast to the robust induction of IFITs (Fig. 2A through C). STAT1 is a key mediator in the induction of ISGs by IFN-α (31). We observed that CH25H induction by IFN-α was almost abolished in STAT1-KO cells, confirming that CH25H is an ISG (Fig. 2D and E). To identify potential regulators of CH25H expression, we focused on microRNAs, key players in gene regulation that control over 30% of human genes (32, 33). Given Dicer’s role in miRNA maturation (34), we used the CRISPR/Cas9 system to generate Dicer-knockout cell lines, and as expected, CH25H expression was significantly upregulated, and its induction by IFN-α was markedly enhanced in the absence of mature miRNAs, at both mRNA and protein levels (Fig. 2F and G). In comparison, other ISGs such as GBP1 and GBP3 also appeared to be regulated by miRNAs, whereas CLEC7A was not (Fig. S1). These results indicate that CH25H expression is regulated by miRNAs.

### IFN-α induces a range of microRNAs

**Fig 3:**
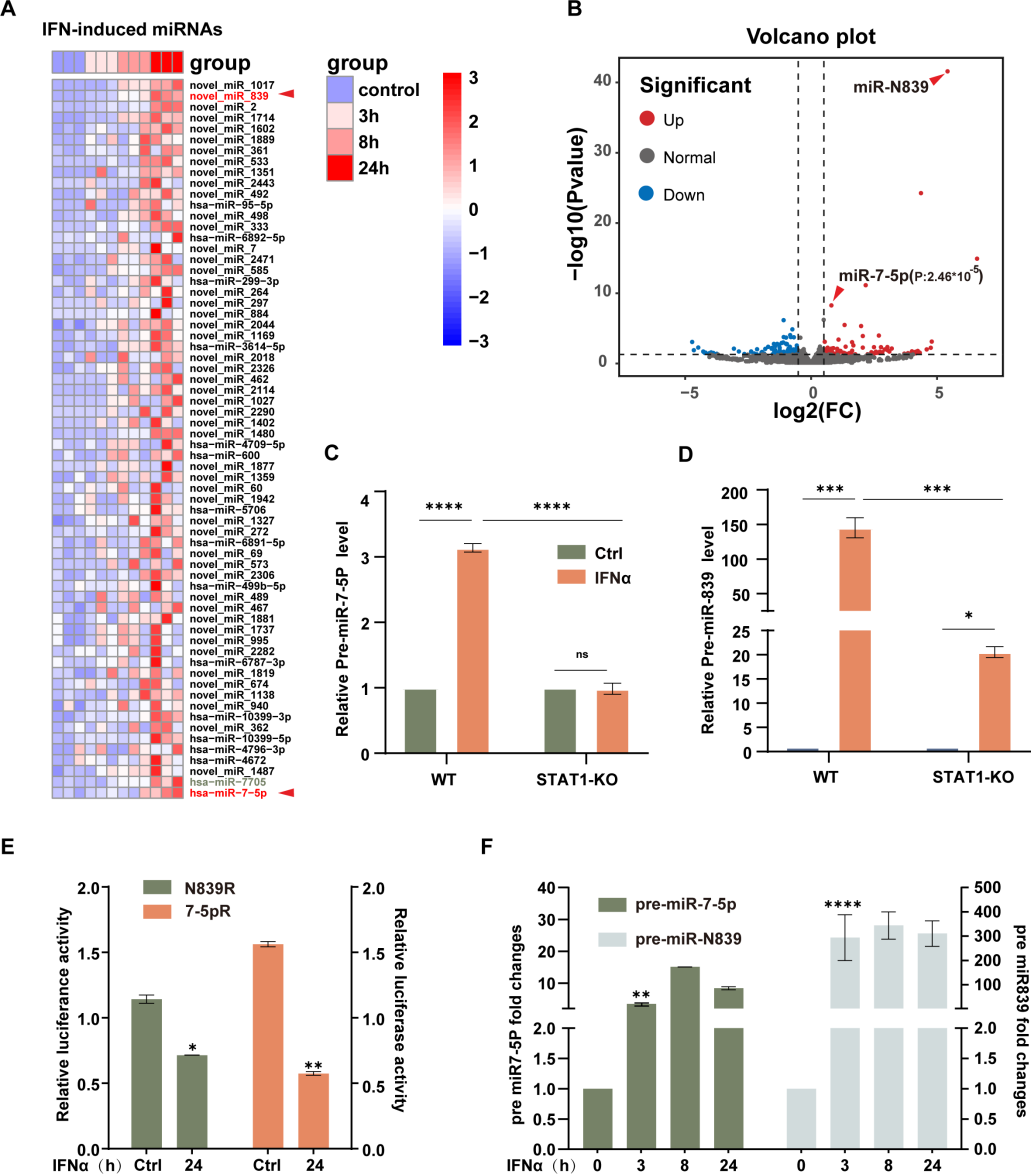
Transcriptomic analysis of microRNAs in IFN-treated HepG2 cells. (**A–B**) Heatmap and volcano plot showing differential expression of microRNAs in response to IFN-α treatment. (**C–D**) HepG2 and STAT1 knockout (KO) cells were treated with IFN-α (20 ng/mL) for 24 h, followed by qPCR analysis to assess the expression levels of pre-miR-7-5p and pre-miR-839. (**E**) A dual-luciferase reporter assay was conducted to evaluate the effect of IFN-α treatment on hsa-miR-7-5p and N839 reporter activity. (**F**) HepG2 cells were treated with IFN-α (20 ng/mL) as indicated, and the expression levels of pre-miR-7-5p and pre-miR-839 were measured by qPCR. Data are presented as mean ± SD from three independent experiments. Statistical significance: *P  <  0.05; **P  <  0.01; ***P  <  0.001; ****P  <  0.0001, ns >0.05.

Using miRNA transcriptomic analysis, we next investigated whether IFN-α-induced microRNAs could target CH25H and modulate its antiviral function. Time-course analysis of IFN-α-stimulated HepG2 cells revealed distinct patterns of microRNA expression. Among the induced miRNAs, hsa-miR-7-5p showed high basal expression, whereas the newly identified novel-miR-839 demonstrated the most robust induction (Fig. 3A and B). Quantitative PCR analysis confirmed a significant induction of both microRNA precursors in wild-type HepG2 cells. However, this response was nearly abolished in STAT1-knockout cells, establishing hsa-miR-7-5p as a canonical interferon-stimulated miRNA (Fig. 3C). In contrast, novel-miR-839 remained inducible in STAT1-deficient cells, suggesting that its expression is not exclusively dependent on STAT1 signaling (Fig. 3D). Further validation using a dual-luciferase reporter assay confirmed their induction (Fig. 3E), and IFN-α treatment also significantly elevated their precursor forms (Fig. 3F). Together, these results demonstrate that IFN-α induces a specific profile of microRNAs in hepatocytes.

### IFN-induced hsa-miR-7705 specifically targets CH25H 3´UTR

**Fig 4:**
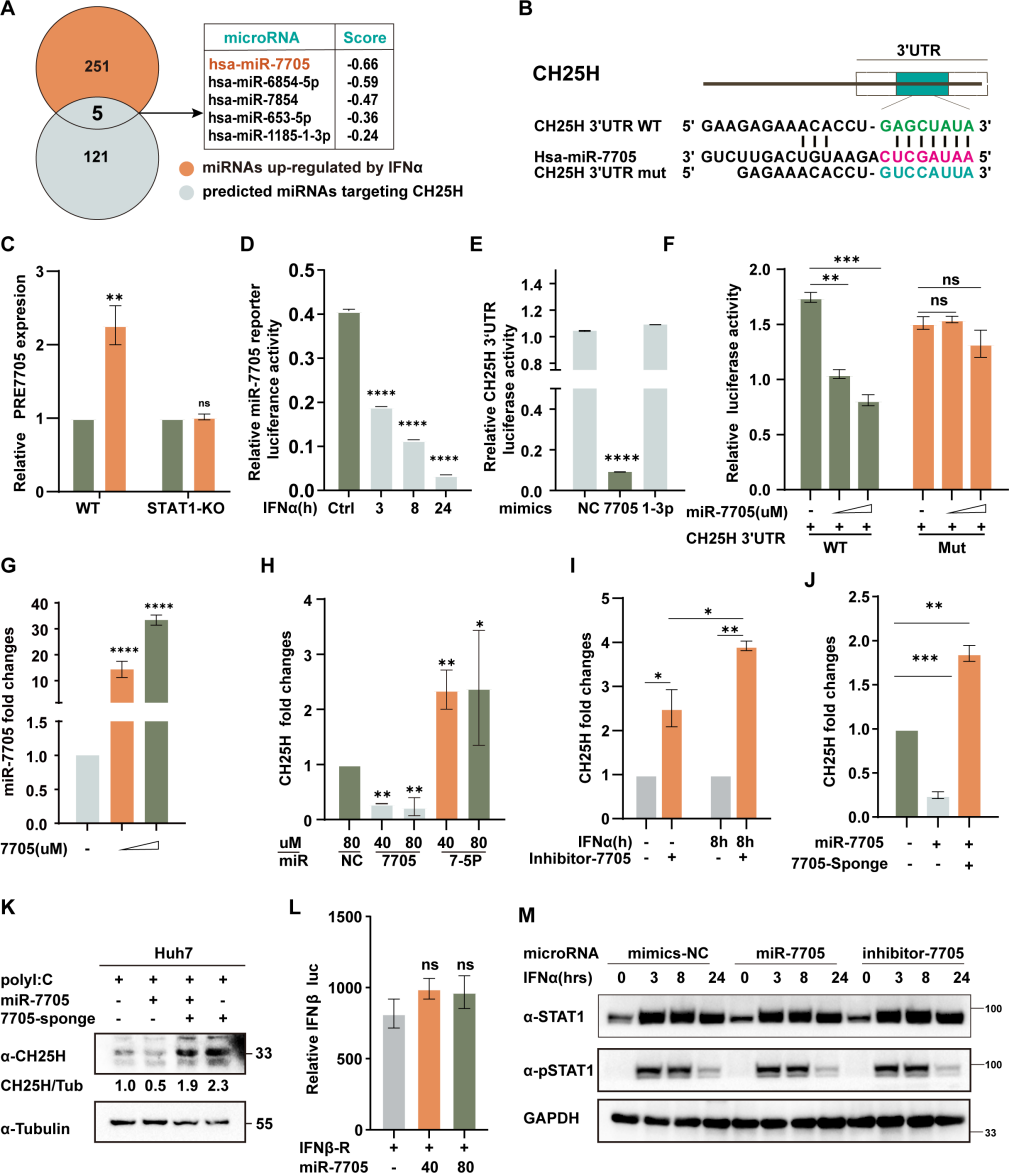
Identification and functional analysis of miR-7705 targeting CH25H. (**A**) Schematic strategy for identifying IFN-induced microRNAs that target CH25H. (**B**) Design of wild-type (WT) and mutant CH25H 3′UTR luciferase reporter constructs. (**C**) HepG2 and STAT1 knockout (KO) cells were treated with IFN-α (20 ng/mL), followed by qPCR analysis to measure pre-miR-7705 expression. (**D–E**) miR-7705 reporter constructs were transfected into HepG2 cells, followed by co-transfection with microRNA mimics (miR-7705, miR-1-3p) or treatment with IFN-α. Dual-luciferase assays were then performed. (**F–G**) HepG2 cells were co-transfected with miR-7705 mimics and either WT or mutant CH25H 3′UTR constructs, followed by dual-luciferase assays to assess miR-7705–mediated regulation. (**H**) HepG2 cells were transfected with miR-7705 or miR-7-5p mimics, and CH25H mRNA expression was analyzed by qPCR. (**I–K**) Various transfection combinations using miR-7705 mimics, inhibitors, or sponges were employed to evaluate their effects on CH25H expression, assessed by qPCR and immunoblotting, as indicated. (**L**) HepG2 cells were co-transfected with an IFN-β luciferase reporter and either miR-7705 mimics or a negative control (NC), followed by luciferase assay to determine the impact of miR-7705 on IFN-β promoter activity. (**M**) HepG2 cells were transfected with miR-7705 mimics, NC mimics, or a miR-7705 inhibitor, as indicated. After IFN-α treatment, whole-cell lysates were collected and analyzed by immunoblotting using antibodies against phospho-STAT1 (pSTAT1), STAT1, and GAPDH. Data are presented as mean ± SD from three independent experiments. Statistical significance: *P  <  0.05; **P  <  0.01; ***P  <  0.001; ****P  <  0.0001, ns >0.05.

To identify microRNAs that potentially regulate CH25H expression, we integrated transcriptomic data with TargetScan predictions. This analysis revealed five miRNAs that were both interferon-inducible and predicted to target CH25H. Among these, hsa-miR-7705 emerged as the top candidate based on predictive targeting scores (Fig. 4A and B). We found that hsa-miR-7705 was specifically upregulated in HepG2 wild-type cells but not in STAT1 knockout cells (Fig. 4C). The induction was further confirmed by an IFN-α-responsive miR-7705 reporter assay (Fig. 4D; Fig. S2). Functional validation using a dual-luciferase assay demonstrated that miR-7705, but not the control miR-1-3p, significantly suppressed luciferase activity linked to the CH25H 3'UTR (Fig. 4E). Critically, although miR-7705 overexpression reduced CH25H 3'UTR luciferase activity, mutation of the predicted binding sites completely abolished this effect (Fig. 4F and G). At the molecular level, miR-7705 mimics, but not hsa-miR-7-5p, effectively reduced CH25H mRNA levels (Fig. 4H). Conversely, inhibition of miR-7705 enhanced IFN-α-induced CH25H expression (Fig. 4I), and a specific miR-7705 sponge reversed the suppressive effects of miR-7705 mimics on CH25H expression (Fig. 4J). These findings were further corroborated at the protein level (Fig. 4K). To exclude potential indirect effects, we demonstrated that miR-7705 overexpression neither affected IFN-β production in a luciferase reporter system (Fig. 4L) nor altered STAT1 activation, as evidenced by unchanged total and phosphorylated STAT1 levels (Fig. 4M). Collectively, these data establish miR-7705 as a direct regulator of CH25H expression through targeting its 3'UTR.

### Hsa-miR-7705 promotes HBV replication

**Fig 5:**
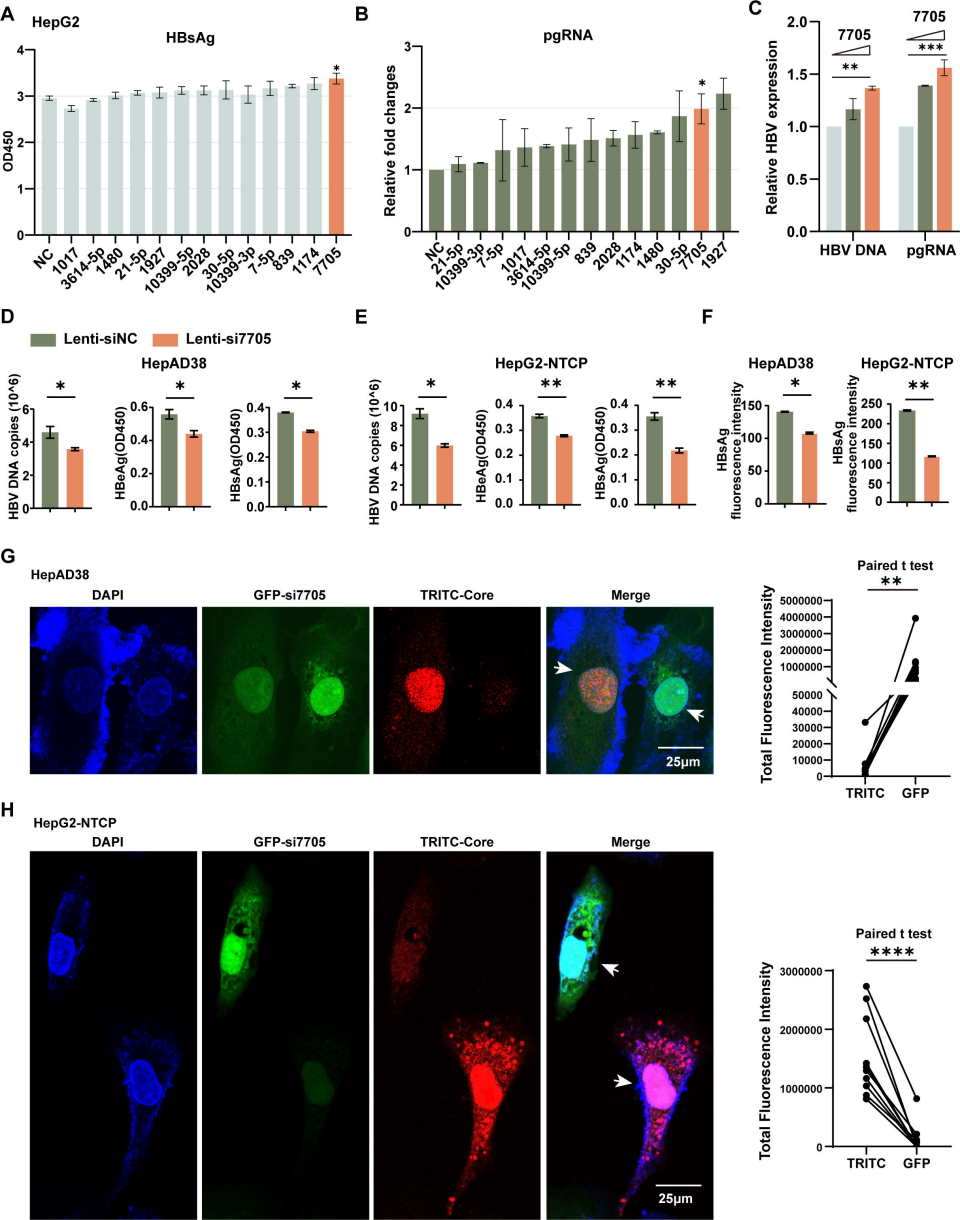
miR-7705 promotes HBV replication. (**A–B**) HepG2 cells were co-transfected with microRNA mimics and pHBV1.2, followed by ELISA analysis to assess HBsAg levels. At 48 h post-transfection, the cells were collected to measure pgRNA levels via qPCR. (**C**) HepG2 cells were transfected with pHBV1.2 and either miR-7705 mimics or negative controls. Cells were collected at 48 h for qPCR analysis. (**D, E**) HepAD38 cells and HepG2-NTCP cells were transduced with lentiviral vectors expressing either miR-7705-targeting siRNA (si-miR-7705) or negative control siRNA (si-NC). HepG2-NTCP cells were infected with HBV for 9 days. Subsequently, the cell culture supernatant was collected to quantify HBV DNA copy numbers by qPCR and measure HBeAg and HBsAg levels by ELISA. (**F**) HBsAg was additionally detected using a novel plasmon-enhanced fluorescence (PEF) platform. (**G, H**) Cells were subjected to immunofluorescence using anti-HBc antibody. The fluorescence intensity of a group of cells selected from 10 positions was analyzed using NIS Elements Viewer. Data are presented as the mean ± SD from three independent experiments. Statistical significance: *P  <  0.05; **P  <  0.01; ***P  <  0.001; ****P  <  0.0001; ns > 0.05.

To investigate the role of the miR-7705-CH25H regulatory axis in HBV replication, we transfected HepG2 cells with microRNA mimics along with HBV plasmids. Among the microRNAs tested, miR-7705 significantly enhanced HBV replication, as evidenced by increased levels of HBsAg, HBeAg, HBV DNA, and pgRNA (Fig. 5A and B; Fig. S3). The enhancing effect was dose-dependent, with higher miR-7705 concentrations resulting in greater increases in HBV replication markers (Fig. 5C). Additionally, we designed siRNA targeting pre-miR-7705 and constructed lenti-simiR-7705 for the knockdown (KD) assay, and miR-7705 knockdown was confirmed in both HepAD38 (a tetracycline-regulated HBV replication cell line) and HepG2-NTCP cells (Fig. S4). HBV DNA copies and HBsAg and HBeAg levels were detected in both HepAD38 and HBV-infected HepG2-NTCP cell lines. As anticipated, all markers were reduced in the miR-7705 knockdown cell lines (Fig. 5D and E). For HBsAg detection, we implemented a novel plasmon-enhanced fluorescence (PEF) platform utilizing thermally annealed gold nanoparticles (TA-GNPs), as reported in the literature (35). The results were also consistent with our ELISA data (Fig. 5F). Interestingly, we used NIS Elements Viewer to select two groups of cells with high GFP fluorescence intensity (Lenti-simiR-7705 transduced HepAD38 cells) or high TRITC fluorescence intensity (HBV-infected HepG2-NTCP cells). The analysis demonstrated that HBc protein expression was significantly lower in GFP-high expressing cells (Lenti-simiR-7705), whereas the cells with high TRITC fluorescence intensity (HBc) showed relatively weaker GFP fluorescence (Fig. 5G and H; Fig. S5C and S6C). No significant differences were observed in the Lenti-siNC control group (Fig. S5A, B, S6A and B). Collectively, these data suggest that miR-7705 acts as an enhancer of HBV infection and replication.

### miR-7705 promotes HBV replication by inhibiting CH25H

**Fig 6:**
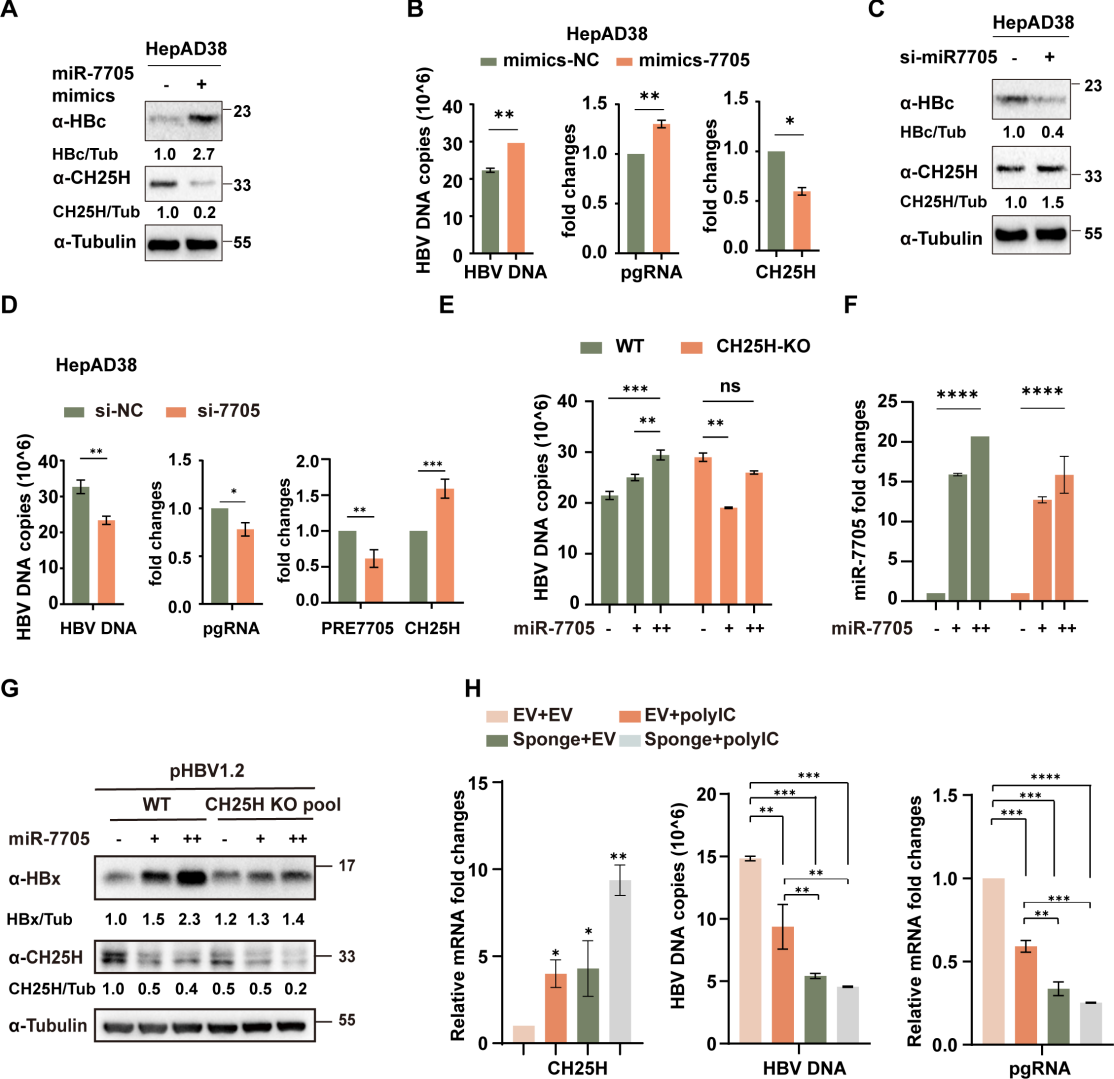
miR-7705 enhances HBV replication by suppressing CH25H (**A, B**) HepAD38 cells were transfected with miR-7705 mimics or negative controls. At 48 h post-transfection, the cells were harvested for (**A**) immunoblotting to evaluate HBc, CH25H, and tubulin protein levels, and (**B**) qPCR to assess CH25H, HBV DNA, and pgRNA expression. (**C, D**) HepAD38 cells were transfected with si-miR-7705 or negative controls. At 48 h post-transfection, the cells were collected for (**C**) immunoblotting of HBc, CH25H, and tubulin, and (**D**) qPCR analysis of pre-miR-7705, CH25H, HBV DNA, and pgRNA levels. (**E–G**) Wild-type (WT) and CH25H knockout (KO) HepG2 cells were co-transfected with pHBV1.2 and miR-7705 mimics to assess HBV DNA copies and miR-7705 expression by qPCR, and (**G**) HBx protein levels by immunoblotting. (**H**) HepG2 cells were co-transfected with a miR-7705 sponge or empty vector along with pHBV1.2, followed by poly(I:C) treatment. Expression of CH25H, pgRNA, and HBV DNA copies was assessed by qPCR. Data are presented as mean ± SD from three independent experiments. Statistical significance: *P  <  0.05; **P  <  0.01; ***P  <  0.001; ****P  <  0.0001, ns >0.05.

In order to validate the role of the miR-7705-CH25H axis in HBV replication. Overexpression of miR-7705 reduced CH25H protein levels and significantly enhanced HBV replication. Conversely, knockdown of miR-7705 by siRNA increased CH25H protein levels, suppressed HBV DNA and pgRNA levels, and reduced HBc protein expression in HepAD38 cells (Fig. 6A through D). Interestingly, in line with our previous report that CH25H inhibits HBx nuclear translocation (10), we discovered that miR-7705 knockdown significantly reduced HBx signaling in the nucleus (Fig. S7). These findings suggest that miR-7705 may function by counteracting CH25H’s inhibitory effects on HBV. To establish CH25H’s role in this pathway, we generated CH25H knockout cells using CRISPR/Cas9 technology. In these cells, miR-7705’s enhancing effect on HBV replication was completely abolished, demonstrating that miR-7705’s pro-viral activity is directly mediated through CH25H (Fig. 6E through G). Although previous research demonstrated that poly(I:C) induces CH25H expression to inhibit HCV replication, our data revealed that poly(I:C) similarly upregulates CH25H expression and suppresses HBV. Importantly, sequestering miR-7705 using a specific sponge further amplified both CH25H expression and its inhibitory effect on HBV (Fig. 6H). Collectively, these findings establish that miR-7705 promotes HBV replication by antagonizing CH25H’s antiviral activity and that targeting miR-7705 enhances CH25H-mediated viral suppression.

### miR-7705 attenuates the antiviral effect of interferon against HBV

**Fig 7:**
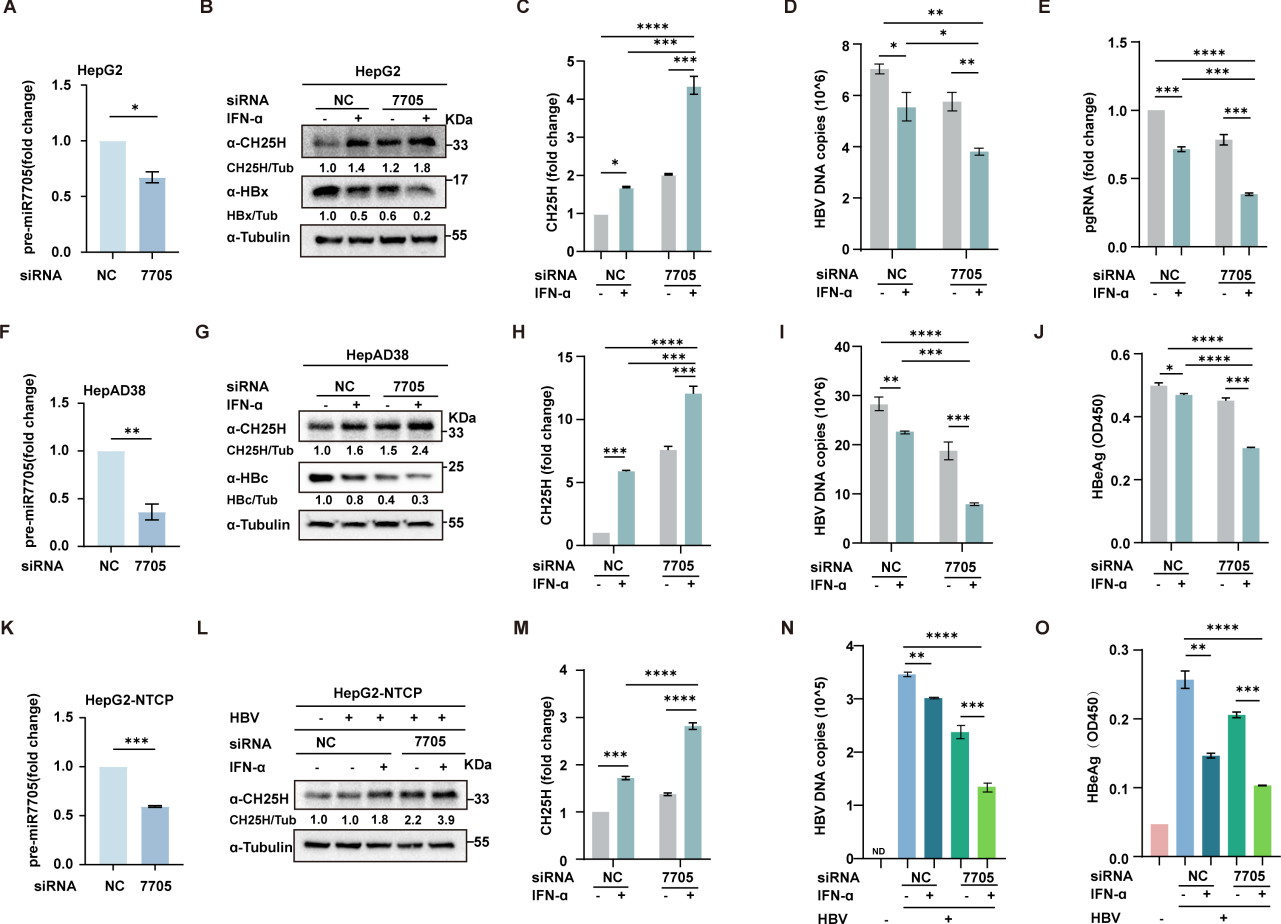
Knockdown of miR-7705 enhances CH25H expression and inhibits HBV infection and replication. (**A**) HepG2 cells were transduced with lentiviral vectors expressing either miR-7705-targeting siRNA (si-miR-7705) or negative control siRNA (si-NC). Knockdown efficiency was validated by qPCR. Cells were then transfected with the pHBV1.2 plasmid. At 24 h post-transfection, the cells were treated with IFN-α (20 ng/mL) for an additional 24 h, after which they were harvested for analysis. (**B**) Western blotting (WB) was performed to evaluate the expression of CH25H, HBx, and tubulin. (**C–E**) qPCR was conducted to assess the mRNA levels of CH25H, HBV DNA copies, and pgRNA, respectively. (**F**) HepAD38 cells were similarly transduced with si-miR-7705 or si-NC lentiviral constructs, and knockdown efficiency was validated by qPCR. (**G**) Cells were cultured for 48 h and then treated with IFN-α (20 ng/mL) for 24 h. Whole-cell lysates were harvested for WB to assess CH25H, HBc, and tubulin expression. (**H–I**) qPCR was used to quantify CH25H levels and HBV DNA copies. (**J**) HBeAg levels in the culture supernatant were measured by ELISA. (**K**) HepG2-NTCP cells were transduced with si-miR-7705 or si-NC, and knockdown efficiency was validated by qPCR. Cells were then infected with HBV or left uninfected, followed by a 9-day culture period. During this post-infection period, cells were treated with or without interferon-α (IFN-α, 20 ng/mL) for 24 h prior to each medium change. (**L**) WB was performed to assess CH25H and tubulin expression. (**M–N**) qPCR was used to quantify CH25H levels and HBV DNA copies, respectively. (**O**) HBeAg levels were evaluated by ELISA. Data are presented as the mean ± SD from three independent experiments. Statistical significance: *P  <  0.05; **P  <  0.01; ***P  <  0.001; ****P  <  0.0001, ns >0.05.

To further elucidate the role of miR-7705 in the interferon (IFN)-mediated anti-HBV response, we used lenti-simiR-7705 for the knockdown (KD) assay. Three experimental models were employed: HepG2 cells transfected with pHBV1.2, HepAD38 cells, and HepG-NTCP cells infected with HBV. Knockdown of miR-7705 was confirmed in all three cell lines (Fig. 7A, F, and K). As anticipated, IFN treatment induced both mRNA (Fig. 7C, H, and M) and protein levels of CH25H, with further upregulation observed following miR-7705 knockdown. In contrast, the kinetics of HBx and HBc protein levels followed an inverse pattern relative to CH25H (Fig. 7B, G, and L). Importantly, IFN-mediated inhibition of HBV replication was markedly enhanced in the miR-7705 KD groups across all three models (Fig. 7D, E, I, J and N and O). This enhancement was corroborated by miR-7705 sponge-mediated blockade (Fig. S8). Collectively, these findings indicate that miR-7705 plays a pivotal role in the IFN-mediated anti-HBV response, likely through the modulation of CH25H.

### miR-7705–CH25H axis broadly modulates RNA and DNA virus replication

**Fig 8:**
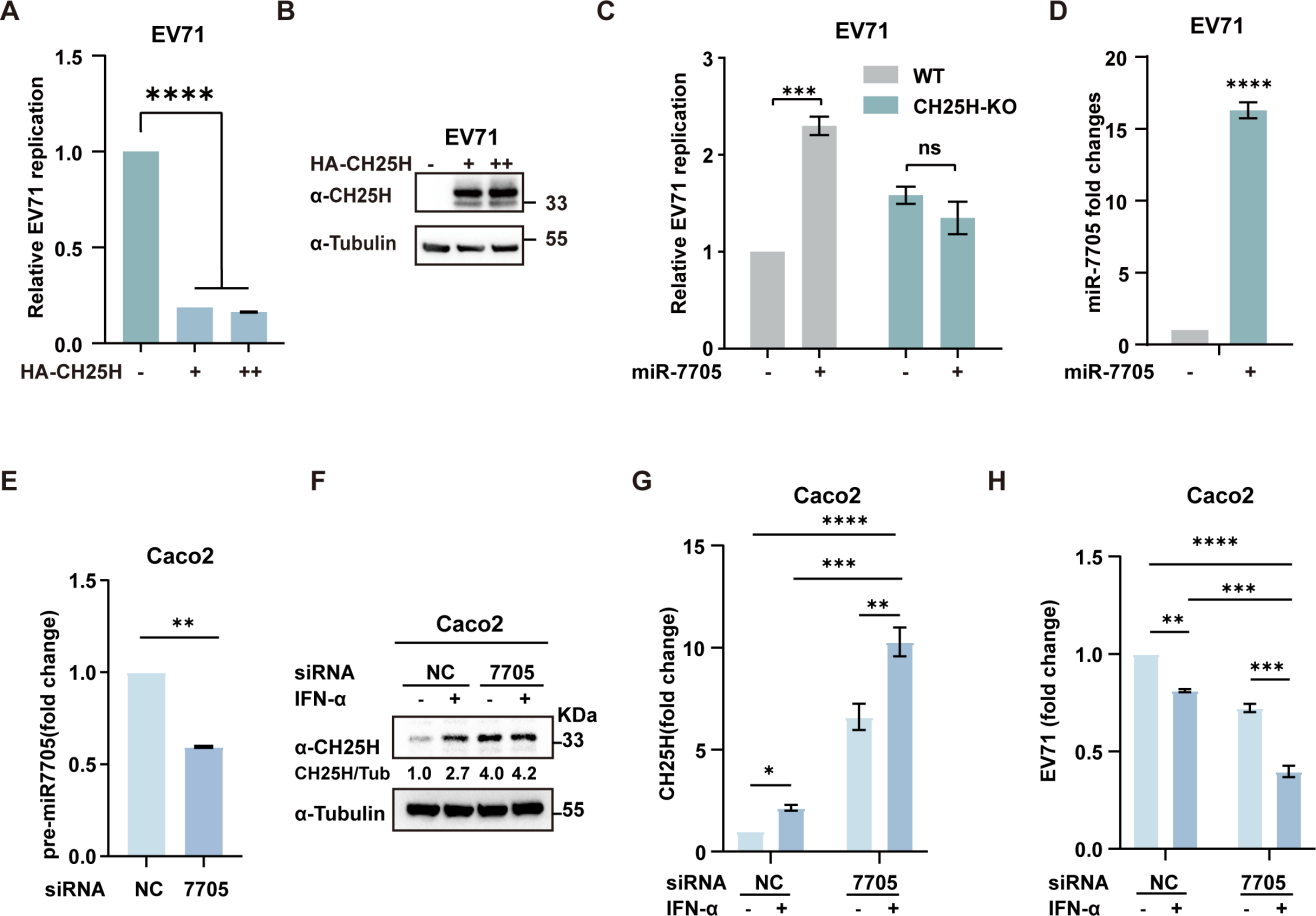
miR-7705 promotes EV71 replication by inhibiting CH25H (**A-B**) HepG2 cells transfected with plasmids expressing HA-tagged CH25H were infected with EV71. EV71 infection was assessed by qPCR (**A**), whereas CH25H protein levels were evaluated through immunoblotting (**B**). (**C-D**) Wild-type (WT) and CH25H-knockout (CH25H-KO) HepG2 cells were transfected with either miR-7705 mimics or negative control mimics, followed by EV71 infection. Virus and miR-7705 levels were then quantified by qPCR. (**E**) Caco2 cells were transduced with lentiviral vectors expressing either miR-7705-targeting siRNA (si-miR-7705) or negative control siRNA (si-NC). Knockdown efficiency was confirmed by qPCR. (**F-H**) The cell lines were infected with EV71. After 24 h, the cells were treated with IFNα (20 ng/mL) for an additional 24 h before harvesting. Western blotting was performed to assess CH25H protein levels, with tubulin used as a loading control (**F**). qPCR analysis was conducted to measure CH25H mRNA expression and EV71 viral RNA levels (**G-H**). Data are presented as mean ± SD from three independent experiments. Statistical significance: *P  <  0.05; **P  <  0.01; ***P  <  0.001; ****P  <  0.0001, ns >0.05.

To evaluate the breadth of the miR-7705–CH25H axis in antiviral defense, we examined its effects on the single-stranded RNA virus EV71, in comparison to the DNA virus HBV. In HepG2 cells infected with EV71, wild-type CH25H significantly suppressed viral replication (Fig. 8A and B). Overexpression of miR-7705, however, enhanced the replication of EV71. Notably, this pro-viral effect was largely abolished in CH25H-knockout cells (Fig. 8C and D), indicating that the ability of miR-7705 to promote EV71 replication—similar to its effect on HBV—is CH25H-dependent. Furthermore, knockdown of miR-7705 in Caco2 cells (Fig. 8E) led to increased CH25H mRNA and protein expression (Fig. 8F and G) and enhanced the antiviral efficacy of interferon (IFN) against EV71, compared with control cells (Fig. 8H). Similarly, the role of miR-7705-CH25H was also confirmed in another RNA virus, CVB3 (Fig. S9). Collectively, these findings highlight that the miR-7705–CH25H regulatory axis acts as a broad-spectrum modulator of viral replication, impacting both RNA and DNA viruses. This underscores the potential of targeting the miR-7705–CH25H axis as a therapeutic strategy against a wide range of viral pathogens.

## DISCUSSION

Interferon (IFN) plays an important role in the treatment of HBV-related diseases (1). However, IFN therapy for chronic HBV infection faces several notable limitations. Although pegylated IFN-α (PEG-IFN-α) can achieve sustained virological control, its efficacy remains suboptimal for many patients due to the development of IFN resistance and significant side effects  (36). These adverse effects often limit patient compliance and therapeutic duration. Moreover, although IFN therapy activates a broad range of interferon-stimulated genes (ISGs), their downstream effects are not uniformly antiviral, and in some cases, they may even contribute to immune dysregulation or cytotoxicity. Previous studies have reported low overall response rates and high discontinuation rates due to adverse effects  (37, 38), underscoring the need for adjunctive strategies to enhance IFN efficacy or minimize its side effects.

Cholesterol 25-hydroxylase (CH25H) is an IFN-inducible enzyme that catalyzes the production of 25-hydroxycholesterol (25HC), a metabolite with broad-spectrum antiviral activity. CH25H has been shown to inhibit a range of viruses, including HCV, Zika virus, and SARS-CoV-2, via mechanisms involving modulation of lipid metabolism and interference with viral entry and replication  (39–41). Despite being classified as an ISG, CH25H is only weakly induced by IFN in hepatocytes  (8), suggesting post-transcriptional repression. One potential explanation is the induction of specific microRNAs (miRNAs) that target CH25H and attenuate its expression.

MiRNAs are critical post-transcriptional regulators of gene expression, with well-documented roles in modulating host-virus interactions (42). For example, miR-122 enhances HCV replication by stabilizing the viral genome (43), whereas HBV suppresses miR-192-3p to promote autophagy and facilitate replication (17). In this study, we identified miR-7705 as a negative regulator of CH25H, directly binding to the 3′ untranslated region (3′UTR) of CH25H mRNA and leading to its downregulation. This interaction attenuates CH25H’s antiviral effects against HBV, as well as other RNA viruses such as CVB3 and EV71. Inhibition of miR-7705 via siRNA or sponge constructs restores CH25H expression and enhances its antiviral function, revealing a novel miRNA-mediated mechanism that limits the efficacy of IFN therapy.

To further evaluate the functional significance of this regulatory axis in the context of IFN treatment, we performed additional experiments (Fig. 7) using three HBV models, including HBV-infected HepG2-NTCP cells. We found that inhibition of miR-7705 significantly potentiated IFNα-induced CH25H expression, both at the mRNA and protein levels, and led to enhanced suppression of HBV replication markers. These findings suggest that the miR-7705-CH25H axis serves as a fine-tuning mechanism that tempers ISG overactivation and may contribute to IFN resistance in hepatocytes.

Although the enhancement of IFN’s antiviral effect following miR-7705 knockdown was moderate (~1.5-fold to 2-fold), this is consistent with the notion that miRNAs serve as modulators rather than on/off switches in antiviral signaling networks. Interestingly, even in Dicer knockout or miR-7705-inhibited cells, CH25H protein levels plateaued despite robust mRNA upregulation, indicating potential translational feedback control or post-translational regulation when CH25H reaches a certain threshold. This highlights the complexity of ISG regulation and points to additional layers of control beyond miRNA repression.

The identification of miR-7705 as a post-transcriptional suppressor of CH25H offers a new therapeutic target to optimize IFN-based antiviral strategies. Similar to how miR-122 inhibitors have been developed to treat HCV infection  (44, 45), miR-7705 inhibitors could be explored to enhance endogenous CH25H expression and improve antiviral efficacy in HBV-infected patients. Given the broad antiviral properties of CH25H and the IFN-inducible nature of miR-7705, this axis may also be relevant in the context of other viral infections, such as influenza or coronaviruses.

In summary, our study demonstrates that miR-7705 negatively regulates CH25H, a key antiviral ISG, and thereby attenuates the IFN-mediated suppression of HBV replication (Graphical abstract). Targeting the miR-7705-CH25H axis could enhance IFN antiviral efficacy and represents a promising direction for the development of adjunctive therapies in chronic viral infections. Moreover, we further observed that miR-7705 is induced by IFN-α in both hepatocytes and non-hepatic cells such as THP1 monocytic cells, and its expression is significantly elevated in HBV-infected individuals (Fig. S10). This suggests that HBV modulates miR-7705 levels, likely as part of its interaction with host immune responses. These findings provide a broader context for understanding how IFN therapy and miR-7705 interplay in chronic HBV infection.

## Data Availability

This study’s plasmids and cell lines are available under a standard material transfer agreement. The miRNA transcriptome sequencing data generated in this study have been deposited in the NCBI GEO database under accession number GSE306696. All other data supporting the findings of this study are included within the article and its supplemental material. Requests for resources should be directed to the lead contact, Guangyun Tan (tgy0425@jlu.edu.cn).

## SUPPLEMENTAL MATERIAL

Graphical abstract jvi.01198-25-s0001.tif Visual depiction of the study findings.

Supplemental material jvi.01198-25-s0002.docx Figures S1 to S10; Tables S1 to S5.

ASM does not own the copyrights to Supplemental Material that may be linked to, or accessed through, an article. The authors have granted ASM a non-exclusive, world-wide license to publish the Supplemental Material files. Please contact the corresponding author directly for reuse.
