# Supplementary figures and images for: Interferon-induced miR-7705 modulates the anti-virus activity of cholesterol 25-hydroxylase
Source: J Virol. 2025 Sep 10;99(9):e01198-25. doi: 10.1128/jvi.01198-25 (PMC12455982; doi:10.1128/jvi.01198-25)

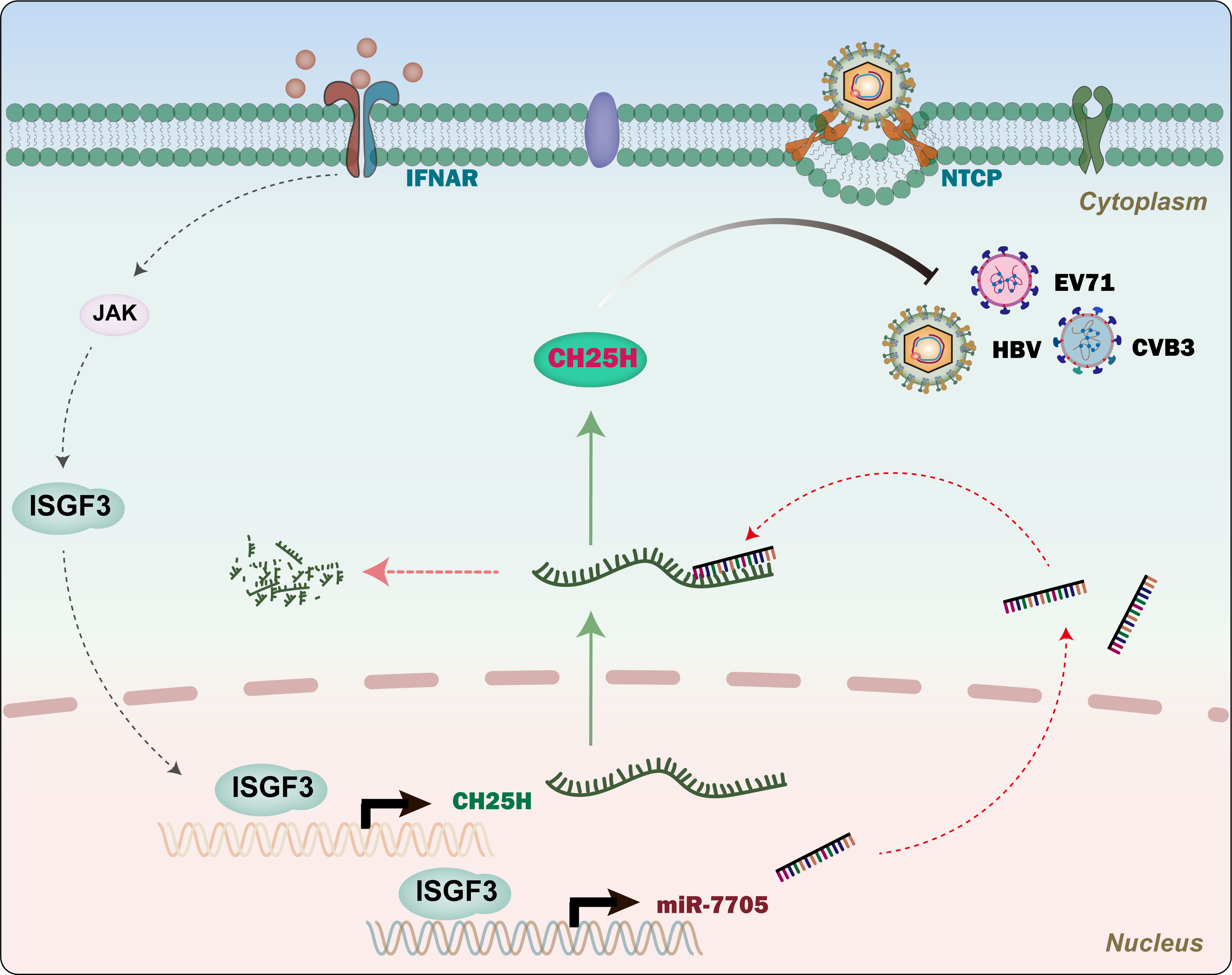

Supplement: Graphical abstract — Visual depiction of the study findings. [file jvi.01198-25-s0001.tif]
