# Supplementary material for: Interferon-induced miR-7705 modulates the anti-virus activity of cholesterol 25-hydroxylase
Source: J Virol. 2025 Sep 10;99(9):e01198-25. doi: 10.1128/jvi.01198-25 (PMC12455982; doi:10.1128/jvi.01198-25)
Supplement: Supplemental material — Figures S1 to S10; Tables S1 to S5. [file jvi.01198-25-s0002.docx]

**Figure S1. DICER knockout enhanced the expression and induction of GBP1/3, but not CLEC7A.** 293T wild-type (WT) and DICER knockout (KO) cells were treated with IFN-α (20 ng/mL) or left untreated, as indicated. Cells were then harvested for qPCR analysis to assess CLEC7A, GBP1, and GBP3 mRNA expression levels. Data are presented as mean ± SD from three independent experiments. Statistical significance: *P < 0.05; **P < 0.01; ***P < 0.001; ****P < 0.0001, ns > 0.05.


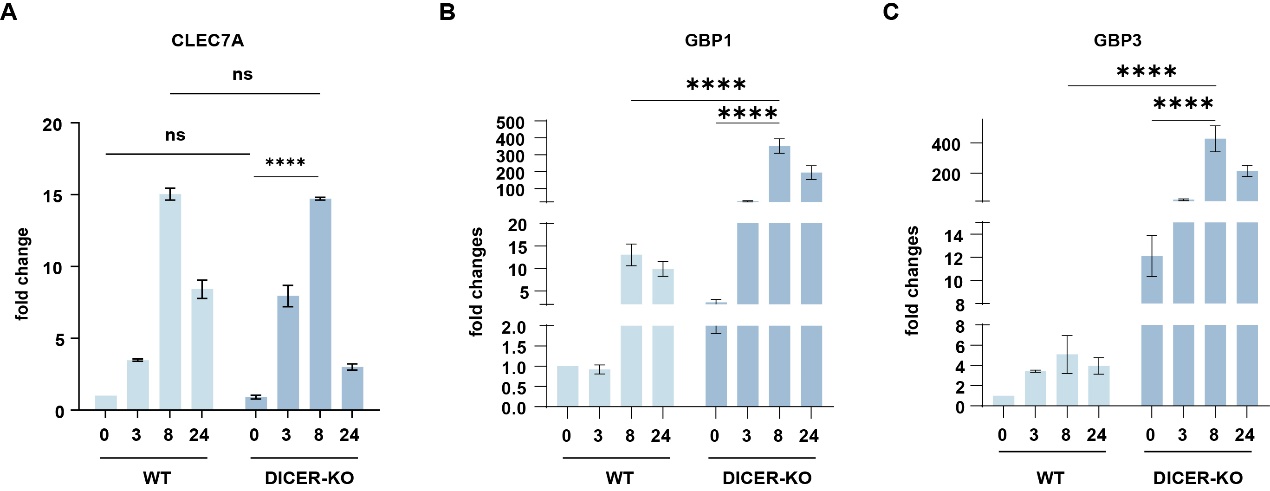


**Figure S2. IFN-α stimulation does not affect the luciferase signal in cells transfected with the pmirGLO empty vector.** HepG2 cells were transfected with the pmirGLO empty vector and cultured for 30 hours. Twenty-four hours prior to harvest, cells were treated with IFN-α (20 ng/mL) as indicated. Dual-luciferase assays were then performed. Data are presented as mean ± SD from three independent experiments. Statistical significance: ns > 0.05.


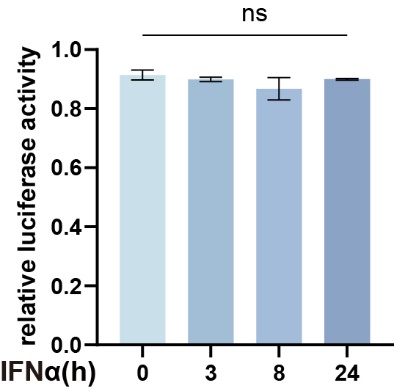


**Figure S3.** **miR-7705 promotes HBV replication.** (A–B) HepG2 cells were co-transfected with miRNA mimics and pHBV1.2, followed by qPCR analysis to assess HBV DNA copies. Supernatants were collected 48 hours post-transfection for HBeAg quantification via ELISA. Data are presented as mean ± SD from three independent experiments. Statistical significance: *P < 0.05; ***P < 0.001.


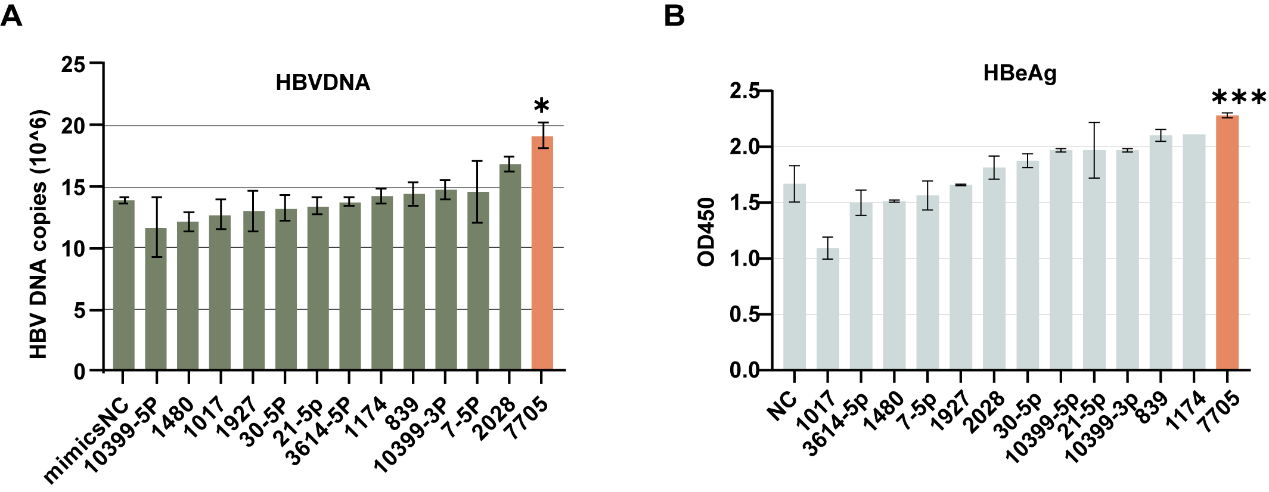


**Figure S4.** **Knockdown of miR-7705 in HepAD38 and HepG2-NTCP cells.** (A–B) HepAD38 and HepG2-NTCP cells were transduced with Lenti-siNC or Lenti-si7705. After 48 hours, quantitative PCR was performed to measure the levels of pre-miR-7705. Data are presented as mean ± SD from three independent experiments. Statistical significance: *P < 0.05; ***P < 0.001.

**
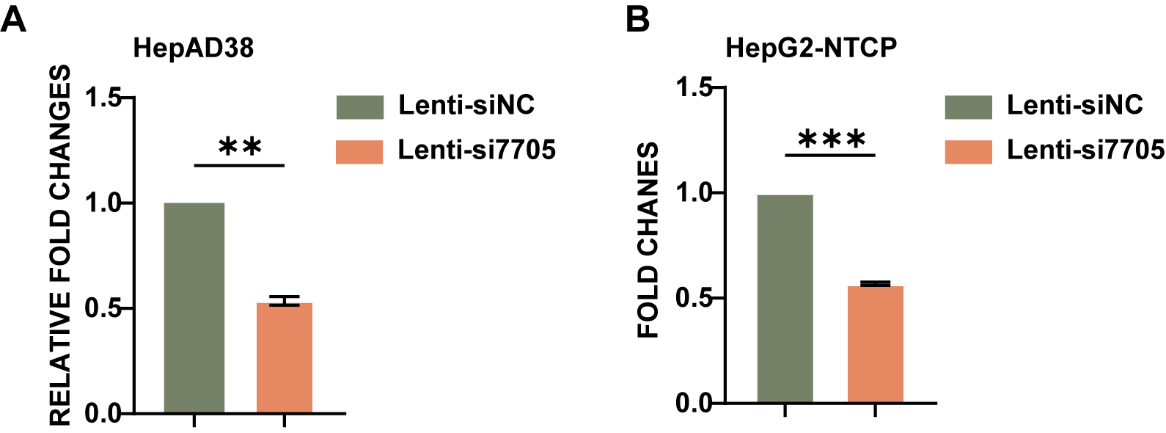
**

**Figure S5. miR-7705 knockdown reduced HBc protein level in HepAD38 cells**

A, C. HepAD38 cells transduced with lentiviral vectors expressing either miR-7705-targeting siRNA (si-miR-7705) or negative control siRNA (si-NC), marked with GFP, were subjected to immunofluorescence using anti-HBc antibody, with GFP fluorescence visible under a fluorescence microscope. B. The fluorescence intensity of a group of cells selected from 10 positions of A was analyzed using NIS Elements Viewer.

**
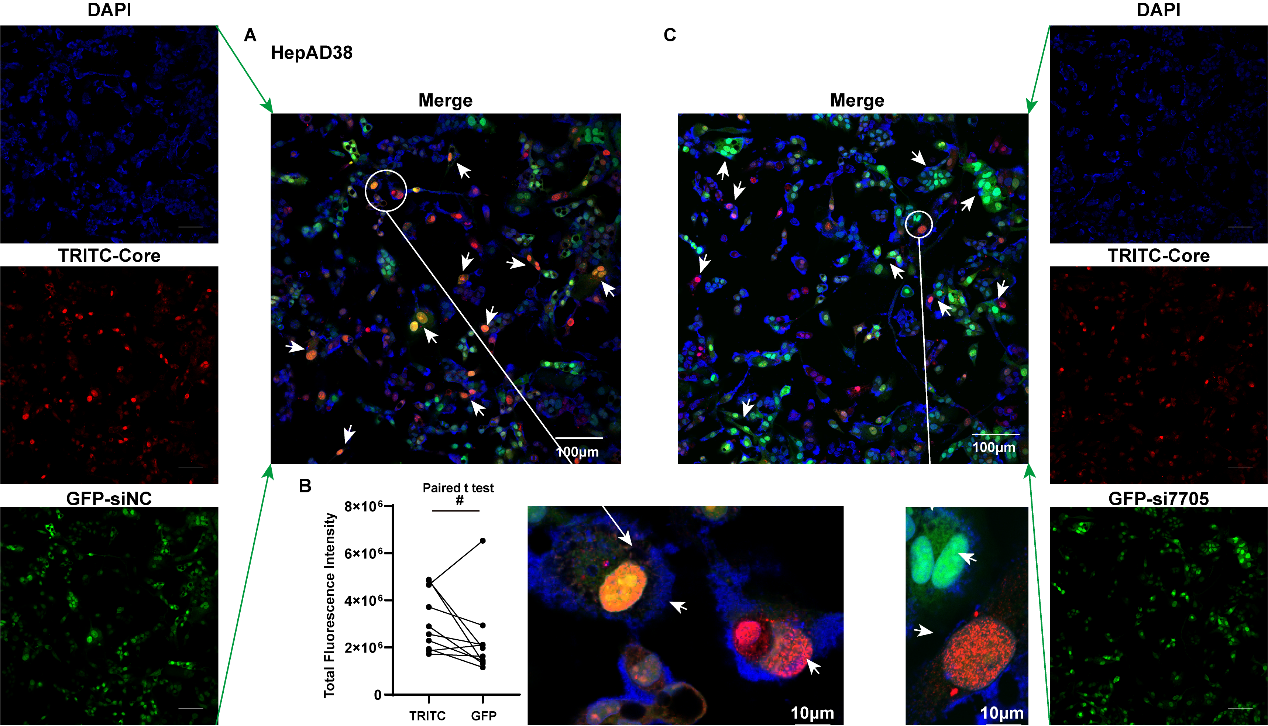
**

**Figure S6. miR-7705 knockdown inhibited HBc protein level in HepG2-NTCP cells**

A, C. HepG2-NTCP cells transduced with lentiviral vectors expressing either miR-7705-targeting siRNA (si-miR-7705) or negative control siRNA (si-NC), marked with GFP, were infected with HBV. Nine days post-infection, cells were subjected to immunofluorescence using anti-HBc antibody, with GFP fluorescence visible under a fluorescence microscope. B. The fluorescence intensity of a group of cells selected from 10 positions of A was analyzed using NIS Elements Viewer.

**
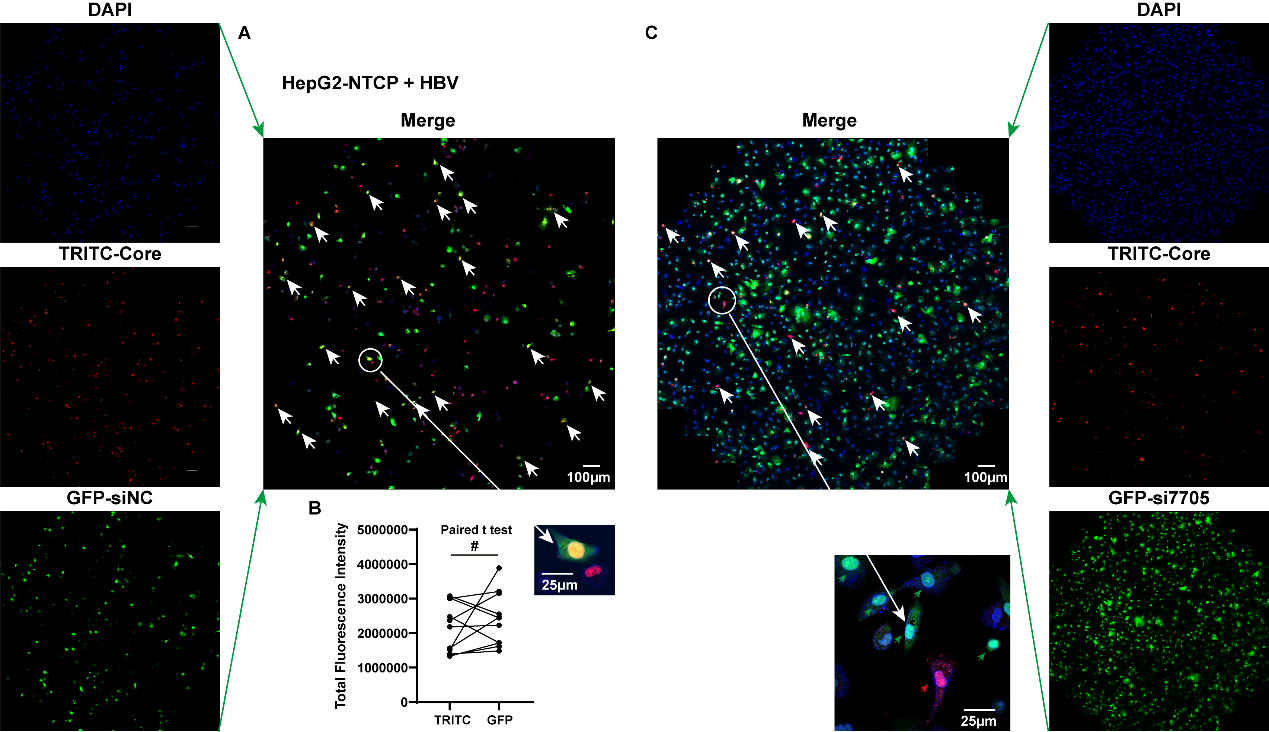
**

**Figure S7. miR-7705 promotes HBx nucleus translocation**

HepG2 cells transduced with lentiviral vectors expressing either miR-7705-targeting siRNA (si-miR-7705) or negative control siRNA (si-NC) were transfected with Flag-HBx. After 36 hours, the cells were washed with PBS and subjected to immunofluorescence using anti-HBx antibody.

**
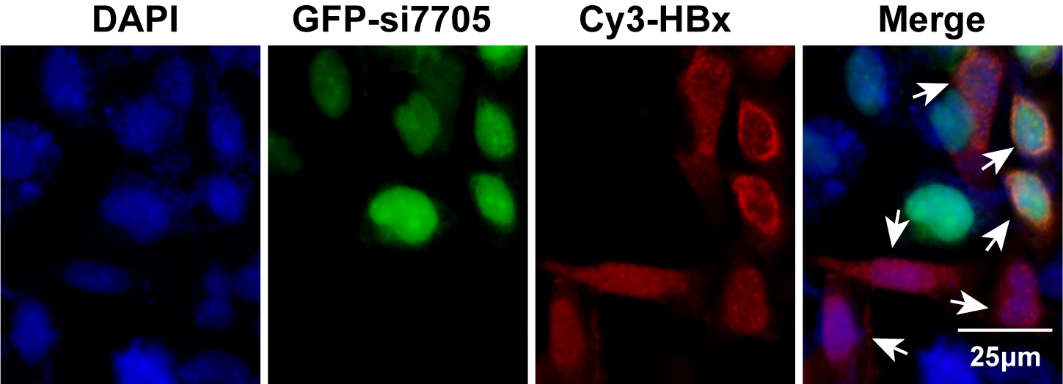
**

**Figure S8.** **miR-7705 sponge enhanced the antiviral effect of Interferon**

(A–B) HepG2 cells were co-transfected with VR-7705-Sponge or VR-Ctrl-Sponge and pHBV1.2. After 24 hours, cells were treated with IFN-α (20 ng/mL) for an additional 24 hours. qPCR was then performed to assess CH25H levels and HBV DNA copies. (C) Supernatants were collected to evaluate HBeAg levels via ELISA. Data are presented as mean ± SD from three independent experiments. Statistical significance: *P < 0.05; **P < 0.01; ***P < 0.001; ns > 0.05


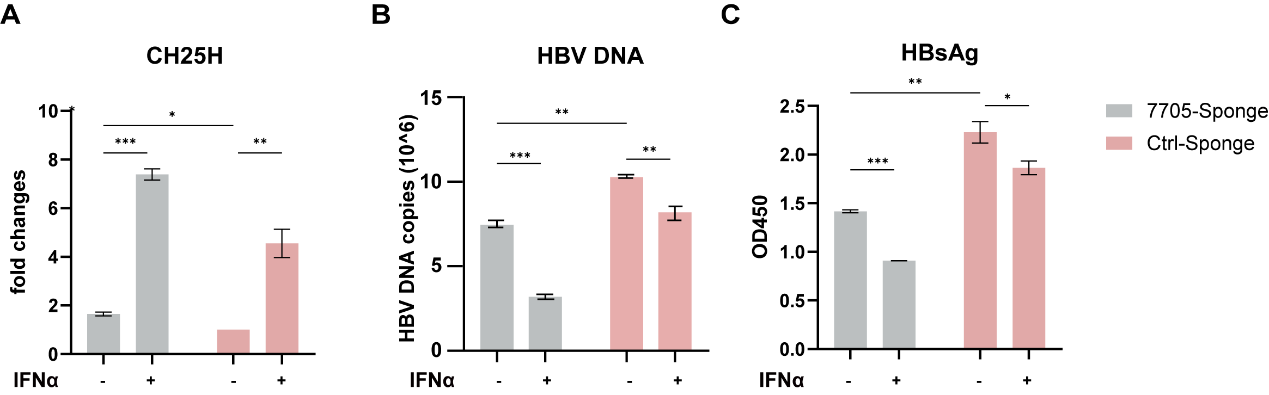


**Figure S9. miR-7705 Promotes CVB3 Replication by Inhibiting CH25H**

(A–B) HepG2 cells transfected with HA-tagged CH25H expression plasmids were infected with CVB3. CVB3 RNA levels were assessed by qPCR (A), and CH25H expression was confirmed by immunoblotting (B). (C–D) Wild-type (WT) and CH25H knockout (CH25H-KO) HepG2 cells were transfected with miR-7705 mimics or negative controls, followed by CVB3 infection. CVB3 and miR-7705 levels were then quantified by qPCR. Data are presented as mean ± SD from three independent experiments. Statistical significance: **P < 0.01; ****P < 0.0001; ns > 0.05.


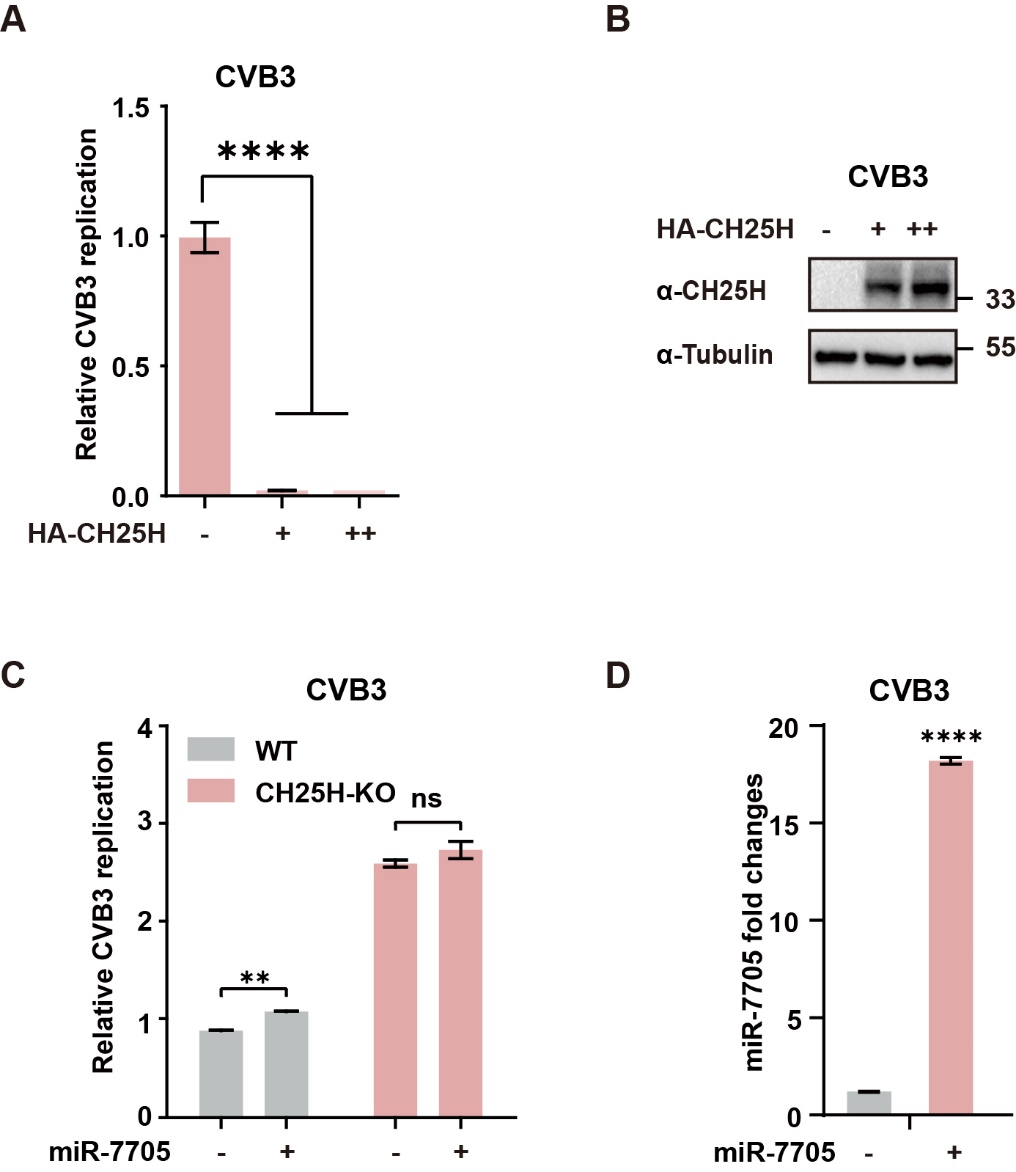


**Figure S10. HBV modulates miR-7705 expression** (A) qPCR quantification of HBV DNA and pgRNA levels in HepAD38 cells before and after TET treatment. (B–C) qPCR analysis of CH25H (B) and pre-miR-7705 (C) expression under the same conditions. (D) Western blot analysis of HBc protein levels, with α-Tubulin used as a loading control. (E) PBMCs were isolated from healthy donors (Ctrl, n = 18) and HBV-infected patients (HBV, n = 28); total RNA was extracted, and pre-miR-7705 levels were quantified by qPCR. (F) qPCR analysis of pre-miR-7705 expression in THP1 cells treated with IFN-α (20ng/mL) for the indicated time points. Data represent means ± SD from three independent experiments. Statistical significance was determined by unpaired two-tailed Student’s t-test: *P < 0.05; **P < 0.01; ***P < 0.001; ****P < 0.0001, ns > 0.05.


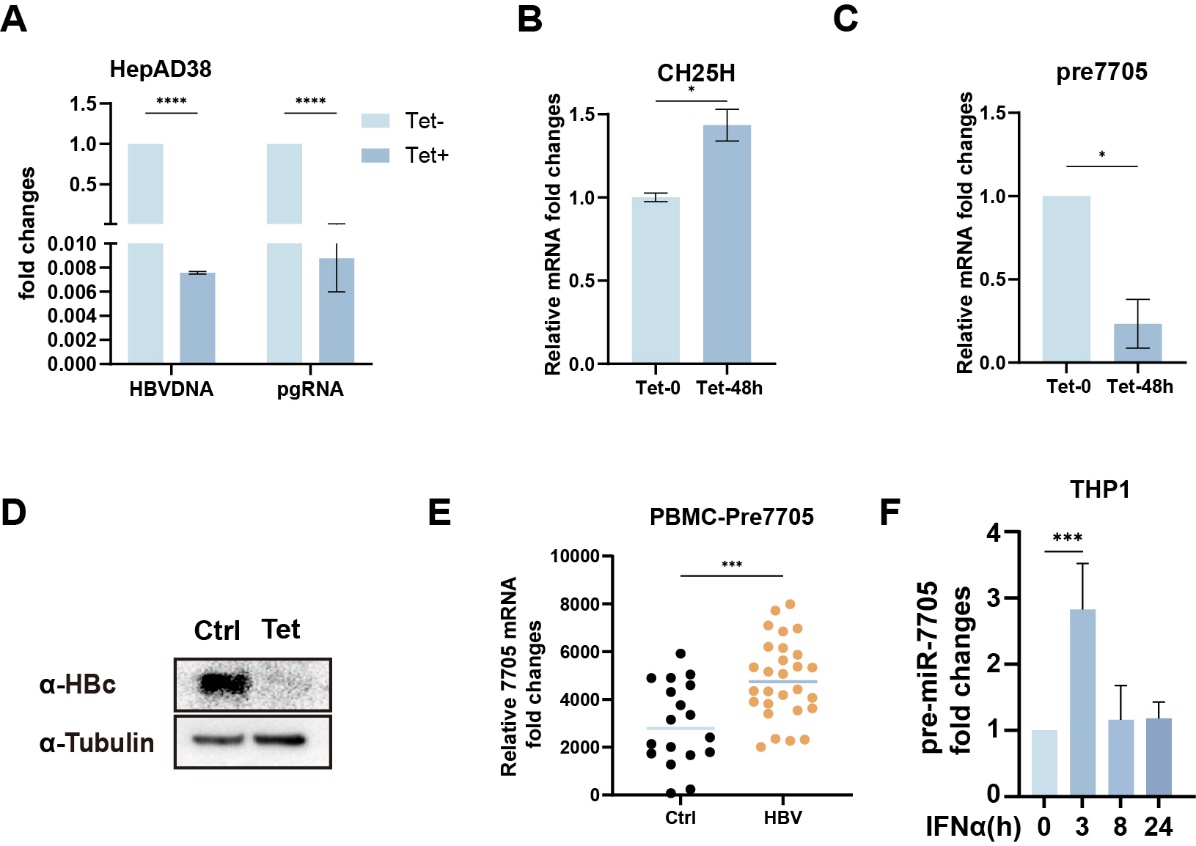


**Supplementary Table 1. Clinical characteristics of HBV-infected patients**

|  |  |  | **HBV-infected patients** | | | | |
| --- | --- | --- | --- | --- | --- | --- | --- |
| No. | Sex | Age (years) | AST (U/L) | ALT (U/L) | HBV DNA (copies/mL) | HBsAg (IU/mL) | HBeAg (S/CO) |
| 1 | F | 39 | 18.3 | 21.5 | 170000000 | 67932.55 | 1805.888 |
| 2 | F | 50 | 18.2 | 14.8 | 740 | >250 | 0.451 |
| 3 | F | 50 | 34 | 32.9 | 3580 | 3117.27 | / |
| 4 | M | 50 | 98.8 | 151.3 | 2180 | 342.4 | 0.451 |
| 5 | M | 57 | 184 | 130 | 505 | >250 | 824.993 |
| 6 | M | 51 | 42.8 | 48.7 | 1280000 | 3736.25 | 23.547 |
| 7 | F | 46 | 57 | 56.9 | 355000 | 49813.76 | 1052 |
| 8 | M | 33 | 33.1 | 76.2 | 33700 | / | / |
| 9 | M | 42 | 27 | 34.7 | 244000 | / | / |
| 10 | M | 43 | 119.7 | 116 | 83500000 | 4978.35 | 984.091 |
| 11 | M | 56 | 25.6 | 20.4 | 282 | 1342.31 | / |
| 12 | M | 70 | 130.6 | 185.3 | 55300 | >250 | 0.42 |
| 13 | F | 51 | 20.8 | 16.7 | 276 | 16008.67 | / |
| 14 | M | 57 | 252.1 | 63.6 | 8420 | 40.55 | / |
| 15 | M | 64 | 53.1 | 33.3 | 126 | >250 | 0.351 |
| 16 | F | 33 | 40.7 | 62.3 | 102 | 72.9 | 0.312 |
| 17 | M | 40 | 52.6 | 101.8 | 2620 | 2477.24 | 0.342 |
| 18 | F | 46 | 228.6 | 347.5 | 21200000 | >250 | 1.741 |
| 19 | F | 41 | 29.2 | 32.6 | 118000 | 2065.64 | / |
| 20 | M | 57 | 63.6 | 252.1 | 8420 | 40.55 | / |
| 21 | F | 29 | 20.9 | 17.1 | 1380 | 343.04 |  |
| 22 | M | 22 | / | / | 121000000 | 79262.71 | / |
| 23 | F | 49 | 14.9 | 3.5 | 9560 | >250 | 0.118 |
| 24 | F | 20 | 71 | 88 | 27000000 | 12951.13 | / |
| 25 | M | 35 | 62 | 45.8 | 69.8 | 4088.35 | / |
| 26 | F | 42 | 684.3 | 534.3 | 7010000 | / | / |
| 27 | M | 42 | 91.2 | 127 | 375 | 1414.16 | / |
| 28 | F | 63 | 55 | 33.5 | 114000000 | >250 | 915.267 |

**Supplementary Table 2. Clinical characteristics of healthy controls**

| No. | Sex | Age (years) | AST (U/L) | ALT (U/L) | HBV DNA (copies/mL) | HBsAg (IU/mL) | HBeAg (S/CO) |
| --- | --- | --- | --- | --- | --- | --- | --- |
| 1 | M | 39 | 16.1 | 16.9 |  |  |  |
| 2 | F | 56 | 59.6 | 46.1 |  |  |  |
| 3 | M | 60 | 18.4 | 26.2 |  |  |  |
| 4 | M | 52 | 19.4 | 19.3 |  |  |  |
| 5 | F | 34 | // | // |  |  |  |
| 6 | F | 34 | // | // |  |  |  |
| 7 | F | 45 | // | // |  |  |  |
| 8 | M | 43 | // | // |  |  |  |
| 9 | F | 51 | // | // |  |  |  |
| 10 | F | 32 | // | // |  |  |  |
| 11 | M | 18 | // | // |  |  |  |
| 12 | M | 23 | // | // |  |  |  |
| 13 | M | 31 | // | // |  |  |  |
| 14 | M | 51 | // | // |  |  |  |
| 15 | F | 46 | // | // |  |  |  |
| 16 | M | 27 | // | // |  |  |  |
| 17 | M | 18 | // | // |  |  |  |
| 18 | F | 26 | // | // |  |  |  |

**Supplementary Table 3. PCR primers**

| **Gene** | **Sequence** |
| --- | --- |
| CH25H 3’UTR  Mut | F: GAGAAACACCTGTCCATTATATTTTTTTAAAGC |
|  | R: GCTTTAAAAAAATATAATGGACAGGTGTTTCTC |
| CH25H 3’UTR  WT | F: TAGCCTCGAGTCTAGTGTGGCTGCGGTGGGTGC |
|  | R: GCAGGTCGACTCTAGTTGACTGCTCAGCGTTAATCTTTC |
| Reporter  miR-7-5P | F: TAGCCTCGAGTCTAGAAACAACAAAATCACTAGTCTTCCATCTAGAGTCGACCTGC |
|  | R: GCAGGTCGACTCTAGATGGAAGACTAGTGATTTTGTTGTTTCTAGACTCGAGGCTA |
| Reporter  Noval-miR-839 | F: TAGCCTCGAGTCTAGAACCTGCACCCTGACACACAAAATCTAGAGTCGACCTGC |
|  | R: GCAGGTCGACTCTAGATTTTGTGTGTCAGGGTGCAGGTTCTAGACTCGAGGCTA |
| Reporter  miR-7705 | F: TAGCCTCGAGTCTAGACAGAACTGACATTCTGAGCTATTTCTAGAGTCGACCTGC |
|  | R: GCAGGTCGACTCTAGAAATAGCTCAGAATGTCAGTTCTGTCTAGACTCGAGGCTA |
| miR-7705 sponge | F: CCGGCAGAACTGAATGCTGAGCTATTCCGGCAGAACTGAATGCTGAGCTATTCCG GCAGAACTGAATGCTGAGCTATTCCGGCAGAACTGAATGCTGAGCTATTCCGGCAGAACTGAATGCTGAGCTATTCCGGCAGAACTGAATGCTGAGCTATTCCGG |
| miR-7705 sponge | R: CCGGAATAGCTCAGCATTCAGTTCTGCCGGAATAGCTCAGCATTCAGTTCTGCCGG AATAGCTCAGCATTCAGTTCTGCCGGAATAGCTCAGCATTCAGTTCTGCCGGAATAGCTCAGCATTCAGTTCTGCCGGAATAGCTCAGCATTCAGTTCTGCCGG |
| CH25H  WT | F: TCGCGGCCGCTCTAGAATGAGCTGCCACAACTGCTC |
|  | R: AGGCGCCTGGTCTAGATCACCGCGCTGGGACAGATG |
| CBFβ | F: TCGCGGCCGCTCTAGAATGCCGCGCGTCGTG |
|  | R: AGGCGCCTGGTCTAGACTAGGGTCTTGTTGTC |
| HMGN2 | F: TCGCGGCCGCTCTAGAATGCCCAAGAGAAAGGCTGA |
|  | R: AGGCGCCTGGTCTAGATCACTTGGCATCTCCAGCAC |
| TRIM31 | F: TCGCGGCCGCTCTAGAATGGCCAGTGGGCAGTTT |
|  | R: AGGCGCCTGGTCTAGATTAGCTTGAAGGAACCTCACAA |
| TRIM5α | F: GAAGAATTCATGGGTTACCCTTATGATGTGCCAGATTATGCCATGGCTTCTGGAATC |
|  | R: GAACTCGAGTCA AGA GCTTGGTGAG |
| TRIM5γ | F: GAAGAATTCATGGGTTACCCTTATGATGTGCCAGATTATGCCATGGCTTCTGGAATC |
|  | R: GAACTCGAGTTATAAGGAGGGGTAAG |
| HBx | F: TCGCGGCCGCTCTAGAATGGCTGCTCGGGTGTGCTGC  R: AGGCGCCTGGTCTAGATTAGGCAGAGGTGAAAAAG |
|  |  |
| TRIM25 | F: TCGCGGCCGCTCTAGAATGGCAGAGCTGTGCCCCCTGG |
|  | R: AGGCGCCTGGTCTAGACTACTTGGGGGAGCAGATGGA |

**Supplementary Table 4. Q-PCR primers**

| **Gene** | **Forward** | **Reverse** |
| --- | --- | --- |
| GAPDH | CGGATTTGGTCGTATTGGG | TCTCGCTCCTGGAAGATGG |
| HBVDNA | GAGTGTGGATTCGCACTCC | GAGGCGAGGGAGTTCTTCT |
| pgRNA | TCTTGCCTTACTTTTGGAAG | AGTTCTTCTTCTAGGGGACC |
| CH25H | CTTTCCGTGGAGGACCACTC | TACGGAGCGAAGTTGCAGTT |
| IFIT1 | GCGCTGGGTATGCGATCTC | CAGCCTGCCTTAGGGGAAG |
| IFIT2 | AAGCACCTCAAAGGGCAAAAC | TCGGCCCATGTGATAGTAGAC |
| IFIT3  EV71-VP1 | AAAAGCCCAACAACCCAGAAT  AGCACCCACAGGCCAGAACACAC | CGTATTGGTTATCAGGACTCAGC  ATCCCGCCCTACTGAAGAAACTA |
| CVB3 | ATGAGACCAGGCTGAATGC | TACTGGTTCTGTGAACTTGC |
| Pre-miR-7705 | ATTGATAACTGAGCAAGG | AAAAGATTCTTATGTCGG |
| Pre-miR-N839 | CCGTATGAATGCCCATCAGC | CACAAAAGCCCTCAGGAACT |
| Pre-miR-7-5P | TTGGATGTTGGCCTAGTTCT | ATGGCAGACTGTGATTTGTT |
| U6 | CTC GCT TCG GCA GCA CA | |
| MiR-7705 | AATAGCTCAGAATGTCAGTTCTG | |
| 3'-adaptor primer | GCG AGC ACA GAA TTA ATA CGA C | |
| miR-7-5P | TGGAAGACTAGTGATTTTGTTGTT | |
| Noval-miR-839 | TTTTGTGTGTCAGGGTGCAGGT | |

**Supplementary Table 5. sgRNA**

| **Gene** | **Forward** | **Reverse** |
| --- | --- | --- |
| DICER sgRNA 1 | CACC GCC AGT GTT TAA ACA GAC GA | AAAC TC GTC TGT TTA AAC ACT GGC |
| DICER sgRNA 2 | CACC GTC CAT CAT GTC CTC GCA TTT | AAAC AAA TGC GAG GAC ATG ATG GAC |
| CH25H sgRNA1 | CACC CAGGCAAAAGCCCACGTATG | AAAC CATACGTGGGCTTTTGCCTG |
| CH25H sgRNA2 | CACC GAGGATATCCAGGACCACGAA | AAAC TTCGTGGTCCTGGATATCCTC |
